# Comparative transcriptome analysis of *Gossypium hirsutum* L. in response to sap sucking insects: aphid and whitefly

**DOI:** 10.1186/1471-2164-14-241

**Published:** 2013-04-11

**Authors:** Neeraj Kumar Dubey, Ridhi Goel, Alok Ranjan, Asif Idris, Sunil Kumar Singh, Sumit K Bag, Krishnappa Chandrashekar, Kapil Deo Pandey, Pradhyumna Kumar Singh, Samir V Sawant

**Affiliations:** 1 CSIR-National Botanical Research Institute, Rana Pratap Marg, Lucknow 226001, India; 2 Department of Botany, Banaras Hindu University, Varanasi 221005, India

**Keywords:** Gossypium hirsutum, Aphids, Whitefly, Transcriptome sequencing, Sap sucking insects, Biotic stress

## Abstract

**Background:**

Cotton (*Gossypium hirsutum* L.) is a major fiber crop that is grown worldwide; it faces extensive damage from sap-sucking insects, including aphids and whiteflies. Genome-wide transcriptome analysis was performed to understand the molecular details of interaction between *Gossypium hirsutum* L. and sap-sucking pests, namely *Aphis gossypii* (Aphid) and *Bemisia tabacci* (Whiteflies). Roche’s GS-Titanium was used to sequence transcriptomes of cotton infested with aphids and whiteflies for 2 h and 24 h.

**Results:**

A total of 100935 contigs were produced with an average length of 529 bp after an assembly in all five selected conditions. The Blastn of the non-redundant (nr) cotton EST database resulted in the identification of 580 novel contigs in the cotton plant. It should be noted that in spite of minimal physical damage caused by the sap-sucking insects, they can change the gene expression of plants in 2 h of infestation; further change in gene expression due to whiteflies is quicker than due to aphids. The impact of the whitefly 24 h after infestation was more or less similar to that of the aphid 2 h after infestation. Aphids and whiteflies affect many genes that are regulated by various phytohormones and in response to microbial infection, indicating the involvement of complex crosstalk between these pathways. The KOBAS analysis of differentially regulated transcripts in response to aphids and whiteflies indicated that both the insects induce the metabolism of amino acids biosynthesis specially in case of whiteflies infestation at later phase. Further we also observed that expression of transcript related to photosynthesis specially carbon fixation were significantly influenced by infestation of Aphids and Whiteflies.

**Conclusions:**

A comparison of different transcriptomes leads to the identification of differentially and temporally regulated transcripts in response to infestation by aphids and whiteflies. Most of these differentially expressed contigs were related to genes involved in biotic, abiotic stresses and enzymatic activities related to hydrolases, transferases, and kinases. The expression of some marker genes such as the overexpressors of cationic peroxidase 3, lipoxygenase I, TGA2, and non-specific lipase, which are involved in phytohormonal-mediated plant resistance development, was suppressed after infestation by aphids and whiteflies, indicating that insects suppressed plant resistance in order to facilitate their infestation. We also concluded that cotton shares several pathways such as phagosomes, RNA transport, and amino acid metabolism with *Arabidopsis* in response to the infestation by aphids and whiteflies.

## Background

Plants are sessile organisms; hence, they are easy target for biotic and abiotic stresses, as they cannot escape from these stresses. One million or more phytophagus insect species use plants as a source of their food. These phytophagus insects obtain nutrition from plants either by chewing or by sucking sap from aerial or underground plant parts. During their long period, approximately 350 million years of co-evolution with plants [1], both insects and plants evolved a variety of different interactions. These interactions can be either positive interactions (insect-mediated pollination and seed dispersion) or negative interactions (insects used plant parts as food). In contrast to the fitness of insects to use plants as a food source, plants have also evolved distinct mechanisms that deal with these interactions [2]. Specialized defense mechanisms in plants protect them from insects. The mechanisms are either inherent constitutive defenses, which include physical barriers such as cell wall and cuticle, or induced defense mechanisms in response to insect attacks [3]. The induced defense mechanisms also include the activation of proteinase inhibitor [4], polyphenol oxidase, chitinases, and so on [5] and the release of secondary metabolites, which attract the parasitoid of attacked insects [3]. The induced defense mechanism has recently been demonstrated to be mediated through SA (Salicylic acid) [6], JA (Jasmonic acid) [7], or ET (Ethylene) pathway [8]. Further, several reports support the fact that there is complex crosstalk between plant hormonal pathways that control the plant responses to wounds, insects, and pathogen attacks [9]. However, detailed genetic regulatory mechanisms that govern plants’ interactions with insects are yet to be fully understood. Sap-sucking insects cause severe damage to both crop plants and glass house-grown plants, in both temperate and tropical regions [10]. The damage caused by the sap sucking pests can be direct as well as indirect. Direct damage occurs due to the removal of nutrients by the feeding of insects on plant phloem, which reduces plant vigor and causes shoot and leaf distortion. A more serious problem is due to the large amount of honeydew that they secrete onto leaves and fruits. The honeydew is colonized by sooty molds. The sooty mold interferes with photosynthesis and may lower the quality of fruits and vegetable harvest. Whiteflies and aphids serve as vectors for plant viruses such as Gemini virus, resulting in the spread of viral diseases [9]. The whitefly *Bemisia tabaci* is known to attack more than 500 species of plants, representing 74 plant families. They are particularly serious threats to crops such as squash, melons, cucumbers, pumpkins, tomatoes, eggplant, potatoes, cotton, and okra. Thus, recently, there has been a keen interest in studying the molecular interaction between sap-sucking insects and plants using microarray studies [3,9,10]. These studies identified the involvement of not only defense-related metabolism but also the genes related to normal cell metabolism such as cell wall modification, water transport, vitamin biosynthesis, photosynthesis, carbon assimilation, and nitrogen and carbon metabolism during aphid attack in different plants such as *Arabidopsis thaliana*, *Apium graveolens*, and sorghum [10-12]. Similarly, the expression of trypsin proteinase inhibitor, lipoxigenase, and xyloglucan-endotransglycoxylase genes was shown to be up-regulated, and rubisco subunit and ubiquitin carrier protein were down-regulated [13] during aphid attack. The difference in the performance of aphids and whiteflies on *Arabidopsis thaliana* mutant *PAD4* indicate that besides some commonality of their interaction with plants, aphids and whiteflies also have a different and unique way of interacting with plants [3]. Cotton is a fiber and an oil-yielding crop that is grown all over the world. Four species of cotton are usually grown worldwide [14]; however, the contribution of *Gossypium hirsutum* L. to the total lint cotton production is maximum worldwide [15]. The productivity of cotton is severely affected by both biotic and abiotic stress worldwide [16]. About 1326 species of insects have been reported to attack cotton plants worldwide [17] and among these species, aphids and whiteflies are one of the major pests for cotton agriculture [18]. Thus, we decided to study the response of cotton at a molecular level in response to the infestation by whiteflies (*Bemisia tabaci* Gennadius) and aphids (*Aphis gossypii* G.) by using Roche’s GS-Titanium pyrosequencing.

## Results

### Transcriptome sequencing of cotton infested with aphids (*Aphis gossypii*) and whiteflies (*Bemisia tabacci*) and de novo assembly

To explore the response of *G. hirsutum* towards sap-sucking pests such as aphids and whiteflies, the leaf transcriptomes of plants infested by aphids and whiteflies were compared. The total data output of transcriptome sequencing was 200.8 Mb in control (C), 222.9 Mb in aphids at 2 h (A2), 231.6 Mb in aphids at 24 h (A24), 297.8 Mb in whiteflies at 2 h (W2), and 244.3 Mb in whiteflies at 24 h (W24) of infestation (Table 1). The coverage length of sequencing was 4X in control, 4.4X in A2, 4.6X in A24, 5.9X in W2, and 4.9X in W24. The quality control and processing of data resulted in 676568 (C), 704185 (A2), 726225 (A24), 894884 (W2), and 795441 (W24) number of high-quality reads with an average length of 296.93 (C), 316.64 (A2), 318.94 (A24), 332.83 (W2), and 307.2 (W24), respectively (Table 1). The high-quality reads were assembled for *de novo* assembly by Roche Newbler (GS-Assembler) software. The contigs produced by assembler were 20249 (C), 19974 (A2), 19307 (A24), 23075 (W2), and 18330 (W24) in number with an average read length of 546.99 (C), 516.62 (A2), 511.26 (A24), 559.03 (W2), and 512.68 (W24), respectively, and unassembled data were considered singletons. The number of singletons were 73724 (C), 70696 (A2), 70310 (A24), 67162 (W2), and 72412 (W24) (Table 2). The size distribution of assembled contigs and singletons is provided in Additional file 1 and Additional file 2. Roche’s GS-Titanium sequence reads discussed in this article can be found in the Genebank (http://www.ncbi.nlm.nih.gov/genbank) of the National Center for Biotechnology Information (NCBI) with accession number SRA049118 (C), SRA049119 (A2), SRA049120 (A24), SRA049121 (W2), and SRA049122 (W24).

**Table 1:** The summary of sequencing data output

**Events**	**Control**	**Aphid (2 h)**	**Aphid (24 h)**	**White Fly(2 h)**	**White Fly(24 h)**
Data output (Mb)	200.8	222.9	231.6	297.8	244.3
Coverage length	(4 X)	(4.4 X)	(4.6 X)	(5.9 X)	(4.9 X)
Average read length	296.93	316.64	318.94	332.83	307.2
High quality read	676568	704185	726225	894884	795441

**Table 2:** The overview annotation of contigs generated from control, aphid 2 h, aphid 24 h, whitefly 2 h, whitefly 24 h and super contigs with TAIR blast and cotton EST blast

**Conditions**	**Control**	**Aphid (2 h)**	**Aphid (24 h)**	**Whitefly (2 h)**	**Whitefly (24 h)**	**Super contigs**
Total Contigs	20249	19974	19307	23075	18330	14810
Number of singletons	73724	70696	70310	67162	72412	18315
Average contig length	546.99	516.62	511.26	559.03	512.68	684.43
**TAIR blast**						
Matched contigs (no.)	14548	13564	13062	16779	12780	7932
Unmatched contigs(no.)	5701	6410	6245	6296	5550	6878
**Cotton EST blast**						
Matched contigs(no.)	9402	9334	9177	10281	8748	14230
Unmatched contigs(no.)	10847	10640	10130	12794	9582	580

### Development of common data set for digital expression analysis

The digital expression analyses of differentially expressed contigs were performed by generating a common data set. All the assembled contigs of different events were again assembled in a single pipeline by using Roche GS-assembler. A total of 14810 contigs were generated with an average length of 684.43 bp (Table 2 and Additional file 3). The transcript per million (TPM) value for each transcript was calculated and normalized against the expression of housekeeping genes (see Methods). The total assembled transcriptome represents 84.7% contigs from C, 82.6% contigs from A2, 82.9% contigs from A24, 86.6% contigs from W2, and 79.6% contigs from W24. The quantitative profiling of transcriptome using DEGseq (R-bioconductor) revealed that 158, 465, 123, and 100 contigs were up-regulated in A2, W2, A24, and W24, respectively; whereas 876, 753, 1013, and 1048 contigs were found to be down-regulated as compared with the control in A2, W2, A24, and W24, respectively (Additional file 4).

### Transcriptional response of cotton toward the infestation of whiteflies was faster as compared with aphids

**Figure 1:**
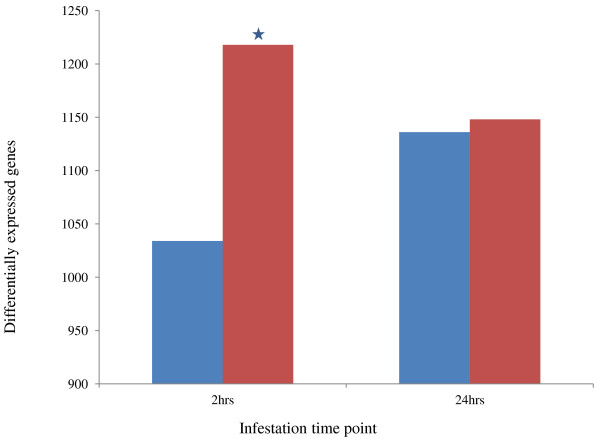
**Number of differentially expressed genes after aphids and whiteflies, 2 and 24 h of infestation, respectively.** Red and blue color bars represent the number of genes induced by the infestation by whiteflies and aphids, respectively.

The cotton plant responds to whiteflies by differentially expressing 1218 transcripts at 2 h of infestation, the number of differential transcripts decreases to 1148 at 24 h of whitefly infestation. In contrast to the number of differentially expressed transcripts in case of aphid infestation which were 1034 at 2 h and increased up to 1136 till 24 h of aphid infestation (Figure 1). The amount of transcripts at W2 was statistically higher (p ≤ 0.002) than A2. Thus, the result indicates that the response of cotton plants toward whiteflies was rather fast, which gets decreased with time; whereas cotton plants respond slower to infestation by aphids and it was similar to response of whiteflies 24 h infestation. In order to find fold-change between aphid- and whitefly-influenced transcript, we checked the average inducibility of these differentially expressed genes and found that fold change was almost similar in all conditions of up regulation as well as 2 h of down regulated genes (Additional file 5); hence, the conclusion based on the number of differentially expressed genes would be meaningful. Further to confirm linearity in expression pattern among the three biological replicate, we have performed microarray with Affymetrix’s Cotton Chip, the result indicates that the correlation between the replicates was as high as 0.97 to 0.98 in control (Additional file 6) and 0.87 to 0.94 in whitefly infested sample (Additional file 7). Thus, the expression variation between replicates were very low and hence our transcriptome of single sample truly reflect the expression profiling of the event circumventing the need of biological replicates.

### Functional annotation of transcriptomes

We compared the transcriptomic data set with the protein database of TAIR 9 using blastx at the e-value of 10^-5^. The number of contigs matched were 14548 (C), 13564 (A2), 13062 (A24), 16779 (W2), and 12780 (W24) (Table 2), and the number of unmatched contigs were 5701 (C), 6410 (A2), 6245 (A24), 6296 (W2), and 5550 (W24) in the respective transcriptomes. The contigs were also queried with the cotton EST database at the e-value of 10^-5^ by using the blastn program. The number of matched contigs in this blast were 9402 (C), 9334 (A2), 9177 (A24), 10281 (W2), and 8784 (W24), and the number of unmatched contigs were 10847 (C), 10640 (A2), 10130 (A24), 12794 (W2), and 9582 (W24) in the respective transcriptomes (Table 2). Super contigs of the common data set were also blasted with both TAIR 9 and cotton ESTs; the unmatched contigs were found to be 6878 in case of TAIR, whereas they were 580 in case of cotton transcripts (Table 2).

### Major changes in transcriptomes in response to infestation by aphids and whiteflies

We analyzed top five genes that were either induced or repressed at 2 h and 24 h of infestation by either aphids or whiteflies (Table 3). The top three genes induced at 2 h and 24 h were found to be common, namely, inositol oxygenase, phosphate translocator, and transketolase; further, phosphate translocator was found to be in the top-induced gene list of infestation by whiteflies at 2 h. Since phosphate translocator, including triose phosphate translocator, was reported to increase the source of carbon in the form of sugar [19], this finding seems to be an important way by which sap-sucking insects increase their sugar concentration in sap. The other two genes uniquely induced in A2 conditions were NADH dehydrogenase and asparginase. The higher expression of asparaginase also suggests the flow of nitrogen source into the sap. Divol et al. (2005) [11] reported that several genes which were involved in nitrate and sugar remobilization in celery, including glutamine synthase, were induced by aphid infestation. Our study also suggested that expression of the transcripts related to the cellular amino acid and nitrogenous metabolism were induced after aphid attack (Additional file 8). The other two highly expressed genes in A24 include RNA binding protein and bHLH type of transcription factor; the induction of these regulatory genes indicates transcriptional reprogramming induced by aphids. RNA binding gene was also induced during W2 infestation (Table 3). In W2, genes such as protein kinase, β-xylosidase 1, and oxidoreductase (DMR 6) were highly induced. The β-xylosidase 1 (AtBxl1) was reported to be involved in secondary cell wall hemicellulose metabolism and plant development [20]. The initial stage of infestation by whiteflies seems to result in changes in cell wall modification by beta-xylosidase 1 (AtBxl1) and signal transduction through protein kinases cascade. During a later phase of infestation by whiteflies, that is, W24, genes such as threonine aldolase, ferric iron binding, 4-hydroxyphenylpyruvate dioxygenase, hydrolase, and adenine phosphoribosyl transferase 1 were highly induced (Table 3). Further, the later stage of infestation by whiteflies also leads to changes in amino acid metabolism by changing the expression of threonin aldolase and 4-hydroxyphenylpyruvate dioxygenase (Table 3). Similarly, hydrolases may also be involved in the hydrolysis of glucosinolate, which gives the cynate and nitril, and these products are reported to be toxic to the attacking herbivores [21]. The down-regulated gene list in A2 and W24 includes DNAJ heat shock N-terminal domain-containing protein. The gene amino acid transmembrane transporter and ethylene-forming gene 1-aminocyclopropane-1-carboxylate oxidase (ACO) were down-regulated in the A2 condition (Table 3). Rerouting of amino acid transportation in response to aphid infestation was reported [22], and the suppression in the amino acid transmembrane transporter was probably linked to infestation by aphids in cotton. Genes such as chlorophyll-binding and copper ion-binding genes were also suppressed during the initial phase of infestation by aphids. Copper-binding genes were also suppressed in A24 (Table 3). Genes such as phosphoenolpyruvate carboxylase 3, DNA repair protein RAD23, and nodulin family protein were suppressed in A24. Phosphlipase D alpha 2 gene, which is involved in wound response, ET, and ABA [23] signaling, was suppressed in the later phase of infestation by both insects. Genes such as serine-type endopeptidase and cellulose synthase 1 were suppressed in W2. Similarly, genes such as plasma membrane intrinsic protein 2A, myo-inositol-1-phostpate synthase 2, and translocon at the inner envelope membrane of chloroplasts 110 were down-regulated in W2 (Table 3). Genes such as plasma membrane intrinsic protein 2A and protease inhibitor were also down-regulated in W24. Thus, the results clearly indicate that infestation by aphids and whiteflies influences changes in transcriptomes in cotton to promote their infestation; whereas the cotton responds to infestation by expressing certain genes or pathways to counteract the herbivorous behavior of these insects.

**Table 3:** Highly up- and down-regulated genes after infestation by aphids and whiteflies in comparison with controls

**Condition**	**Contig No.**	**TAIR Ids**	**Fold change**	**TAIR description**
**C-A2-up**	contig02851	AT1G14520	37.34	Inositol oxygenase
	contig01769	AT2G25520	19.42	Phosphate translocator
	contig03435	AT3G60750	17.94	Transketolase
	contig00969	ATMG00513	12.76	NADH dehydrogenase subunit 5
	contig02659	AT3G16150	9.27	L-asparaginase
**C-A2-down**	contig02161	AT5G23240	27.69	DNAJ heat shock N-terminal domain-containing protein
	contig00717	AT1G77380	26.99	Amino acid transmembrane transporter
	contig10876	AT1G05010	15.4	1-aminocyclopropane-1-carboxylate oxidase
	contig10706	AT3G54890	14.61	Chlorophyll binding
	contig04440	AT1G20340	14.48	Copper ion binding
**C-A24-up**	contig02851	AT1G14520	15.14	Inositol oxygenase
	contig03435	AT3G60750	11.08	Transketolase
	contig01769	AT2G25520	9.6	Phosphate translocator
	contig07116	AT4G29060	9.2	RNA binding
	contig00064	AT2G46510	8.58	ABA-inducible bhlh-type transcription factor
**C-A24-down**	contig04440	AT1G20340	52.4	Copper ion binding
	contig06734	AT1G52570	36.21	Phosphlipase d alpha 2
	contig00123	AT3G14940	36.03	Phosphoenolpyruvate carboxylase 3
	contig01183	AT5G38470	30.06	DNA repair protein RAD23
	contig00536	AT3G01930	27.03	Nodulin family protein
**C-W2-up**	contig00417	AT3G46290	21.24	Protein kinase
	contig08081	AT5G49360	14.68	Beta-xylosidase 1
	contig01769	AT2G25520	14.01	Phosphate translocator
	contig07116	AT4G29060	13.73	RNA binding
	contig03153	AT5G24530	13.62	Oxidoreductase
**C-W2-down**	contig00067	AT1G76140	41.13	Serine-type endopeptidase
	contig03873	AT4G32410	34.46	Cellulose synthase 1
	contig09854	AT3G53420	33.83	Plasma membrane intrinsic protein 2A
	contig07201	AT2G22240	25.01	Myo-inositol-1-phostpate synthase 2
	contig06820	AT1G06950	21.61	Translocon at the inner envelope membrane of chloroplasts 110
**C-W24-up**	contig02059	AT3G04520	24.43	Threonine aldolase 2
	contig11258	AT5G01600	22.58	Ferric iron binding
	contig01632	AT1G06570	9.68	4-Hydroxyphenylpyruvate dioxygenase
	contig00742	AT5G20250	7.26	Hydrolase
	contig02349	AT1G27450	7.08	Adenine phosphoribosyl transferase 1
**C-W24-down**	contig06734	AT1G52570	71.6366	Phosphlipase d alpha 2
	contig04440	AT1G20340	70.3543	Copper ion binding
	contig09854	AT3G53420	66.1615	Plasma membrane intrinsic protein 2A
	contig13210	AT4G12530	45.8687	Protease inhibitor
	contig02161	AT5G23240	40.1503	DNAJ heat shock N-terminal domain-containing protein

### Expression of defense-related transcripts in response to infestation by aphids and whiteflies

Next, we examined the expression of genes reported to be involved in plant defense in response to various pathogens and insects (Additional file 9). We identified that several kinases were down-regulated in infestation by aphids and whiteflies, which includes Enhanced Disease Resistance 1 (EDR1), MAP kinase 6, MAP kinase 16, and cell wall-associated kinase 5. The roles of these kinases are very well reported in the literature in pathogen-induced plant immunity [24]. Interestingly, the involvement of MPK6-mediated phosphorylation and an increase in the stability of ACS (1-aminocyclopropane-1-carboxylic acid synthase) leads to the production of a high level of pathogen elicitor-induced ET [25] response, which is reported. In our study, ACS 5 was suppressed at W24; MPK6 was suppressed in A2, A24, and W24 (Additional file 9); and ET-forming enzyme 1-aminocyclopropane-1-carboxylate oxidase was suppressed in A2 (Table 3). Thus, the suppression of ASC5, MPK6, and 1-aminocyclopropane-1-carboxylate oxidase (ACO) during W24 and A2 indicates the insect-mediated suppression of the ET pathway. Among the highly up-regulated genes are CBSX1 − cystathionine beta-synthase (CBS) family protein, which was reported to be involved in cysteine amino acid metabolism and also in cell redox maintenance [26]. Another gene that was highly up-regulated during these insect infestations was MLO1 representing mildew resistance locus 1; this gene was reported to be involved in the susceptibility of plants toward fungal pathogens [27]. Further, the involvement of callose synthase in response to pathogen attack and wounding was reported [28], and the involvement of glucan synthase in callose deposition and aphid resistance [29] was recently reported. We identified that a homologue of ATGSL10 representing glucan synthase was up-regulated in all the events; whereas homologues of ATGSL08 and ATGSL12 genes representing glucan synthase were down-regulated at most of the infestation time points, especially during the later phase of infestation (Additional file 9). The differential expression of callose synthase may be considered a strategy of compatible interaction that is used to prevent the plugging of the creation of pores by these insects [30]. We identified that senescence-associated gene 18 (SAG18) was up-regulated in all the events (Additional file 9), indicating a probable strategy of channelizing the flow of free amino acids formed due to the breakdown of leaf proteins during senescence [31]. Further, the expression of Ca^2+^-binding DND2 [32] and CNGC2/DND1 was down-regulated in all the events (Additional file 9); this may lead to an increase in the influx of free Ca^2+^ to sieve elements to plugged damaged sieve elements due to sap-sucking insects and prevent the loss of phloem sap [28]. Other genes representing the wall-associated kinase were highly induced during the initial phase of infestation. Similarly, in the differential expression of these cell walls homeostasis management related genes, other genes related to protein folding and cell membranes were also differentially expressed after infestation; for example, the expression of prefoldin 3 and 5 was up-regulated, whereas prefoldin 6 was down-regulated in infestation by insects. Further, membrane proteins such as SGR3, which represented Syntaxin/t-SNARE and SYP61-syntaxin of 61 family proteins of plants, were down-regulated in A2, A24, and W24 during infestation by these insects (Additional file 9). Interestingly, we identified that one of the major key proteins involved in microRNA biogenesis, that is, the AGO4-argonaute family protein, was found to be up-regulated in all the events, and the role of argonaute in plant immunity was recently reported [33]. Phenylalanine ammonia-lyase-2, the first enzyme involved in the phenylpropanoid pathway, was found to be up-regulated in W2 and W24. Similarly, the role of cytochrome P450 in the various biotic and abiotic stresses is well discussed [34]. We identified that the P450 genes family member CYP86A8 was induced in infestation by both aphids and whiteflies. The expression of the GTPase gene, which is also known as *enhanced disease resistance 3*, was induced in A24 and W2; whereas its expression was down-regulated in A2 and W24. Similarly, the expression of pathogenesis-related 4 possessing chitinase binding activity was induced in A24 and W2; whereas its expression was suppressed in A2. Further, we identified the expression of NPR1-like protein 4, which was reported to be involved in plant resistance [35], was down-regulated in A2 and W24. Interestingly, a homologue of Arabidopsis NAC domain containing protein 2/ ATAF1, which is a negative regulator of plant resistance [36], was down-regulated in the later phase of infestation by aphids and whiteflies. Plant WRKY DNA-binding transcription factors are involved in plant pathogen interactions [37]. We identified that the expression of WRKY 33 was enhanced in all the events (Additional file 9). However, the expression of WRKY 21 was down-regulated in A2 and W24; whereas WRKY 20 was down-regulated in A2, A24, and W24. WRKY 1 was down-regulated in A2, A24, and W2; WRKY 35 was down-regulated in W24; and WRKY 3 was up-regulated in all the cases. Thus, our results suggest that aphids and whiteflies interact with cotton plants via complex molecular interactions involving several pathogenesis-related genes and pathways.

### Oxidative stress

Oxidative radicals play an important role in plants during various stresses, including the biotic stress such as insect infestation [38]. Thus, we checked the expression of genes that are involved in the scavenging of oxidative radicals (Table 4). Glutathione is a major ROS (Reactive oxygen species) scavenger in plants; we observed the expression of enzymes involved in glutathione synthesis, namely*,* glutathione peroxides in A2 and W24, ascorbate peroxidase (APX6) [39] in whitefly insect-infested leaves were down-regulated (Table 4). Further, catalases are the H_2_O_2_ and other ROS detoxifying enzymes produced at the site of ROS/H_2_O_2_ production [40]. We observed a decrease in the expression of catalase1 in all the four events, namely, A2, A24, W2, and W24; further, the expression of catalase 2 was decreased in W24, and a decrease in the level of catalases indicates an increase in H_2_O_2_ level during the infestation by sap-sucking insects. We further observed a decrease in the expression of superoxide dismutase family protein T5P19.1 in A24 and W24; copper chaperon for SOD (Superoxide dismutase) in all the events; copper/zinc superoxide dismutase3 (CSD3) in W24; copper/zinc superoxide dismutase1 (CSD1) in A2, W2, and W24; and Fe superoxide dismutase 3 (FSD3) in A24 and W24 (Table 4). Thus, our results indicate that a decrease in the expression of the ROS scavenging enzyme may enable an increase in the concentration of ROS and H_2_O_2_, which are directly toxic to insects [38] and drive fast peroxidase-mediated oxidative cross-linking of structural proteins in the cell wall [41].

**Table 4:** Expression pattern of reactive oxygen species scavenger genes

**TAIR ID**	**Contig ID**	**Control_log2_TPM**	**Aphid2_log2_TPM**	**Fold change_A2_down**	**Annotation**
AT4G11600	contig11662	6.18	3.85	5.03	Glutathione peroxides
AT1G12520	contig03507	5.58	3.75	3.56	Copper chaperon for SOD
AT1G20630	contig00463	7.79	6.07	3.30	Catalase1
AT1G08830	contig03179	7.04	5.89	2.22	Copper/Zinc Superoxide dismutase1, CSD1
		**Control_log2_TPM**	**Aphid24_log2 _TPM**	**Fold change_A24_down**	
AT5G23310	contig03661	4.62	2.64	3.96	Fe Superoxide dismutase 3, FSD3
AT1G12520	contig03507	5.58	4.22	2.56	Copper chaperon for SOD
AT1G20630	contig00463	7.79	6.71	2.11	Catalase1
AT3G56350	contig03718	5.93	4.92	2.01	Superoxide dismutase family protein T5P19.1
		**Control_log2_TPM**	**Whitefly2_log2_TPM**	**Fold change_W2_down**	
AT1G12520	contig03507	5.58	0.00	47.82	Copper chaperon for SOD
AT4G32320	contig02518	6.32	4.55	3.40	Ascorbate peroxidase APX6
AT1G08830	contig03179	7.04	5.41	3.11	Copper/Zinc Superoxide dismutase1, CSD1
AT1G20630	contig00463	7.79	6.67	2.18	Catalase1
		**Control_log2_TPM**	**Whitefly24_log2_TPM**	**Fold change_W24_down**	
AT1G12520	contig03507	5.58	0.00	47.82	Copper chaperon for SOD
AT4G32320	contig02518	6.32	4.26	4.15	Ascorbate peroxidase APX6
AT1G08830	contig03179	7.04	4.83	4.63	Copper/Zinc Superoxide dismutase1, CSD1
AT1G20630	contig00463	7.79	6.00	3.45	Catalase1
AT5G23310	contig03661	4.62	2.73	3.72	Fe Superoxide dismutase 3, FSD3
AT3G56350	contig03718	5.93	4.69	2.36	Superoxide dismutase family protein T5P19.1
AT4G35090	contig00365	8.36	7.31	2.08	Catalase2
AT5G18100	contig13140	4.54	3.31	2.34	Copper/Zinc Superoxide dismutase3
AT4G11600	contig11662	6.18	4.83	2.55	Glutathione peroxides

### GO classification and mining of the changes in the functional class after attacks by sap-sucking insects

We used *Arabidopsis thaliana* model for ‘GO’ annotation of our transcriptome data. Differentially expressed genes of each event in comparison to the control were analyzed and functionally categorized based on three GO categories at p-values ≤ 0.05, which were performed by using singular enrichment analysis (SEA) of agriGO tool (http://bioinfo.cau.edu.cn/agriGO/). Only the molecular functions (F) and biological process (P)-related GO categories containing more than 4% genes of agriGO annotation were selected for further analysis (Additional file 8). These results showed that the differentially expressed transcripts were involved in various metabolic and cellular processes. The result showed that the major transcriptomic reprogramming in aphids happens in the late phase of infestation and most proportion of these categories were suppressed at later phase of infestation by both insects (Additional file 8). The major groups of up-regulated genes in aphid infestation belong to carboxylase, hydrolase, structural moleculer activity, and stress response by various signals and pathogens. Cellular catabolic and transporter related transcript were suppressed during aphid infestation. Further, the early up-regulated genes also shared the pathways belonging to a developmental or reproductive structure (Additional file 8). We observed that the later phase of infestation by aphids leads to the up-regulation of transcripts related to secondary metabolic processes such as phenyl propanoid biosynthesis, flavanoids, and aromatic compounds (Additional file 8). During the later phase of aphid infestation, leads to the suppression of transcript for phosphorus metabolic process, macromolecule metabolic process including post translational protein modification, RNA metabolic process, nitrogen compound metabolic process, transcript related to purin binding as well as ATP and adenyl nucleotide binding genes (Additional file 8). The result indicates the involvement of secondary metabolic pathways in infestation control of aphids, especially in the later phase. In addition, the later phase of aphid infestation (A24) also showed the up-regulation of genes belonging to amino acid and aromatic compound metabolism. We also noticed a decline in the expression of genes belonging to transporter activity in A2, and a significant number of genes belonging to pyrophosphatase and hydrolases of acid anhydrides are down-regulated in infestation by aphids; however, hydrolases of esters were up-regulated in A2. The transcriptomic reprogramming in response to whiteflies similar to infestation by aphids showed the up-regulation of several genes belonging to stress, response to signals and pathogens (Additional file 8). Interestingly, there was no major secondary metabolic pathway reprogramming in case of infestation by whiteflies in contrast to infestation by aphids (Additional file 8). Another contrasting difference was that many of the transporter activities were up-regulated in case of whiteflies (Additional file 8). Interestingly, a significant proportion of up-regulated genes belonged to transcription regulators, indicating that a later phase of infestation by whiteflies induces transcriptional reprogramming (Additional file 8).

### The response of cotton plants toward aphids and whiteflies shares with hormonal and other biotic stresses

**Figure 2:**
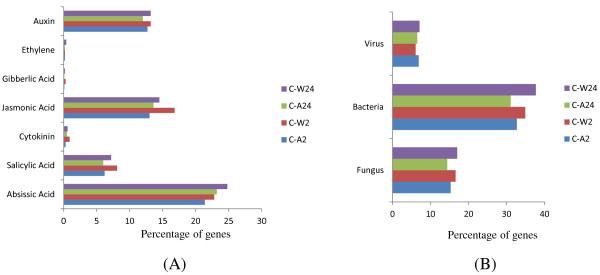
Genevestigator analysis of differentially expressed genes (A) Phytohormonal-responsive transcripts; (B) Fungal viral, and bacterial-responsive transcripts.

The members of genes responding to different hormonal pathways were obtained by querying transcriptome data to the Genevestigator database (https://www.genevestigator.com/gv/plant.jsp). We observed that transcripts responding to abscisic acid (ABA) were increased during the later phase of infestation by both aphids and whiteflies (Figure 2A). Transcripts responding to Jasmonic acid and salicylic acid were relatively higher in whitefly (W2) attack which was decreased with due course of infestation (W24). Similarly, transcripts responding to SA was higher during intial infestation of whiteflies (W2) which was in agreement with previous report where author suggests induction of SA pathways during the attack of whitefly on *Arabidopsis *[3]. We also observed that JA responsive transcript were suppressed during time course of whitefly infestation while there expression were enhanced in aphid infestation. These result support the previous report that inspite some commonalities in infestation mode of these insect, plant deals with them in different ways. JA- and SA-mediated induction of plant defense in response to insect infestation was indicated [3]. We have identified the expression of OPR3 (oxophytodienoate-reductase), which is involved in the JA biosynthesis, and the development of plant defense [42] was up-regulated in A24 and W2 but down-regulated in A2 and W24 (Additional file 9). The role of ACX (acyl-CoA oxidase) genes involved in JA biosynthesis was reported earlier [43]. We found that among the five family members of acyl-CoA oxidase (ACX), four of them were differentially expressed in our experiment (Additional file 9). ACX1 gene was up-regulated in W2 and down-regulated in A24; ACX2 was up-regulated in A24, W2, and W24; ACX3 was down-regulated in all except W2; and ACX4 was down-regulated in all cases except W24. Similarly, expression of genes such as Jasmonate Resistant 1 (JAR1) was up-regulated in W2 (Additional file 4). JAR1 encodes a JA amino acid synthetase that is involved in conjugating jasmonic acid to Ile. The result indicates the involvement of these pathways in the later phase of infestation management by plants. A decrease in the expression of lipoxygenase I (LOX1) and LOX2 in initial phase of infestation of these insects and a decrease in the expression of TGA2 in plants infested by aphids and whiteflies showed insect-mediated suppression of plant defense, which facilitated the compatible infestation of these insects (addional file 4). Transcripts related to ethylene and Giberlic acid was almost similar in all conditions but a significant increase is seen the the transcript related to W2 in cytokinin responses. We next examined the common genes with that of bacterial, viral or fungal infestation. Intrestingly response of cotton to both whitely and aphid show strinking similarity with bacterial response as compare to fungus and virus (Figure 2B). The transcripts similar to bacterial response were increased in whiteflies during later phase of infestation (W24), similar trend was also observed in case of transcript similar to fungal response. The percentage of transcript similar to virual response were found to be almost similar in all conditions. The result showed differential expression of wound induced protein (WIN2), heat shock proteins (Additional file 4). Interestingly the transcript of ferritin is highly induced during whitefly infestation (Additional file 4). These insects also influenced the expression of RNAi machinarry. The ferritin is used to homeostatsis of Fe in cytoplasm of cell. Recently, Kieu et al. [44] reported that Fe is necessory for proliferation of bacterial disease in *Arabidopsis* plant. The induction of ferritin after whiteflies infestation, raised interesting question wheather it is in favour of plant or insect, which need to be address in future. A decreased in the expression of non-specific lipase, which is involved in JA biosynthesis during infestation [45], shows the insect mediated suppression of JA pathway (Additional file 4). Similarly, an increase in the expression of downy mildew-resistant 6 (DMR6) (Additional file 10A), which is a positive regulator of susceptibility to fungi [46], also indicates a probable mechanism that induces a favorable response by insects in host plants (Additional file 4). Further, we have screened the pathogenic organism that also influenced the expression of these insect (aphid and whitefly) infestation-responsive transcripts by the help of Genvestigator (Additional file 11). Among them, fungi such as *Alternaria brassicicola, Botrytis cinerea, Blumeria graminis, Erysiphe cichoracearum, E .orontii, Golovinomyces cichoracearum, Phytophthora infestans*, and *P. paraistica*; bacteria such as *Escherichia coli* and *Pseudomonas syringae*; and viruses such as cabbage leaf curl virus (CalCUV) and turnip mosaic virus (TuMV) were found.

### Different biological pathways operating during infestation by aphids and whiteflies

The differentially expressed transcripts compared with the control for each event were analyzed for the identification of biological pathways that were enriched using KOBAS (http://kobas.cbi.pku.edu.cn/home.do). During initial phase of infestation of these insects, citrate cycle (TCA cycle), lysine degradation, alpha-Linolenic acid metabolism, protein processing related transcripts were enriched in aphid infested plant while transcript of fructose, mannose, and sulfur metabolism were enriched in whitefly infested plants (Table 5). Transcript of pathways like pentose phosphate, sesquiterpenoid and triterpenoid biosynthesis were enriched in both aphid and whitefly infested plant during initial phase (Table 5). Interestingly we obsereved that at later phase of whitefly infestation, transcript of amino acid metabolic pathways of glycine, serine, threonine, alanine, aspartate, glutamate, valine, leucine, isoleucine and histidine were enriched (Table 5). We also observed that whitefly infestation also influence the expression pattern of transcript related to secondary metabolic pathway of flavonoid biosynthesis and vitamin metabolism specially ascorbate at later phase. Transcripts of fatty acid, porphyrin and chlorophyll metabolism were uniquely enriched in whitefly infested cotton plant while in aphid infested cotton plant transcript related to pantothenate and CoA biosynthesis, proteasome, galactose, arginine and proline, butanoate, endocytosis were enriched. Transcript of pathways related to peroxisome and phagosome were enriched in later phase of infestation of both insects. Transcript related to fatty acid elongation, circadian rhythm, brassinosteroid biosynthesis, branched dibasic acid metabolism were enriched during later phase of aphid infetstation. Expression pattern of transcript related to photosynthesis specially carbon fixation were significantly influenced by infestation of these insects (Table 5). The transcripts of different pathways like carotenoid biosynthesis, glyoxylate and dicarboxylate metabolism were enriched in A2, W2 and W24 while beta-Alanine, pyruvate, and glutathione metabolism related transripts were enriched in A2, A24 and W24. Transcripts related to glycolysis and gluconeogenesis pathways were enriched at A2 and W24.

**Table 5:** The KOBAS analysis of differentially expressed genes of aphid and whitefly infestation-responsive genes

**Event**	**KOBAS pathway**	**P-value**
Aphid 2 h	Glyoxylate and dicarboxylate metabolism	0.00043
	Carbon fixation in photosynthetic organisms	0.0032
	Citrate cycle (TCA cycle)	0.0045
	Photosynthesis - antenna proteins	0.0054
	beta-Alanine metabolism	0.0064
	Glutathione metabolism	0.0066
	Lysine degradation	0.007
	Pyruvate metabolism	0.0073
	Pantothenate and CoA biosynthesis	0.013
	Glycolysis / Gluconeogenesis	0.015
	Proteasome	0.017
	Galactose metabolism	0.02
	alpha-Linolenic acid metabolism	0.024
	Arginine and proline metabolism	0.026
	Butanoate metabolism	0.028
	Ribosome	0.038
	Pentose phosphate pathway	0.041
	Sesquiterpenoid and triterpenoid biosynthesis	0.043
	Protein processing in endoplasmic reticulum	0.043
	Endocytosis	0.045
	Carotenoid biosynthesis	0.048
Aphid 24 h	Ribosome	0.00000000021
	Proteasome	0.000077
	Carbon fixation in photosynthetic organisms	0.00015
	Pyruvate metabolism	0.00038
	Glyoxylate and dicarboxylate metabolism	0.0034
	beta-Alanine metabolism	0.0092
	Photosynthesis	0.01
	Pantothenate and CoA biosynthesis	0.018
	Fatty acid elongation	0.018
	Peroxisome	0.018
	Endocytosis	0.02
	Circadian rhythm	0.029
	Glutathione metabolism	0.034
	Butanoate metabolism	0.035
	Phagosome	0.036
	Brassinosteroid biosynthesis	0.0372
	Arginine and proline metabolism	0.039
	C5-Branched dibasic acid metabolism	0.043
	Galactose metabolism	0.048
C-W2	Ribosome	0.00000000061
	Porphyrin and chlorophyll metabolism	0.0011
	Photosynthesis - antenna proteins	0.0062
	Sesquiterpenoid and triterpenoid biosynthesis	0.0079
	Fructose and mannose metabolism	0.0089
	Carotenoid biosynthesis	0.014
	Photosynthesis	0.023
	Carbon fixation in photosynthetic organisms	0.027
	Pentose phosphate pathway	0.032
	Sulfur metabolism	0.036
	Fatty acid metabolism	0.038
C-W24	Glycine, serine and threonine metabolism	0.000087
	Photosynthesis - antenna proteins	0.0002
	Ascorbate and aldarate metabolism	0.0004
	Porphyrin and chlorophyll metabolism	0.00061
	Carbon fixation in photosynthetic organisms	0.001
	Photosynthesis	0.0012
	Fatty acid metabolism	0.0016
	Carotenoid biosynthesis	0.0043
	Pyruvate metabolism	0.0067
	Phagosome	0.0069
	Alanine, aspartate and glutamate metabolism	0.0075
	Peroxisome	0.0091
	Glutathione metabolism	0.013
	Photosynthesis	0.013
	Flavonoid biosynthesis	0.018
	Glyoxylate and dicarboxylate metabolism	0.019
	Histidine metabolism	0.026
	Glycolysis / Gluconeogenesis	0.034
	Metabolic pathways	0.035
	Biosynthesis of secondary metabolites	0.036
	Valine, leucine and isoleucine biosynthesis	0.043
	beta-Alanine metabolism	0.045

### The differentially expressed transcriptomes of cotton in response to aphids and whiteflies share many commonalities with those of *Arabidopsis thaliana* L

**Figure 3:**
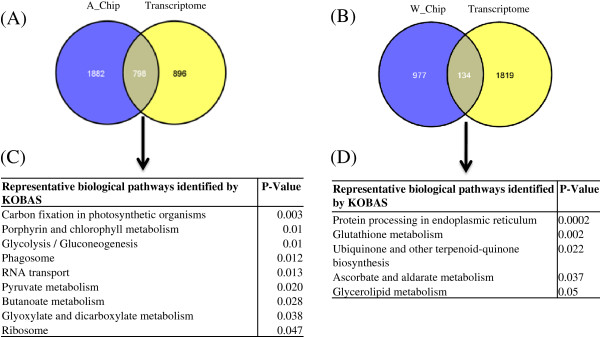
**Venn diagram showing the common and unique genes present in the public domain (GSE5525; GSE6516), aphid-induced (A_Chip), and whitefly induced (W_Chip) with aphid-infested transcriptome (A) and whitefly-infested transcriptome (B) of cotton.** KOBAS analysis of common genes of aphid (**C**) whitefly (**D**) infested public microarray data set and our transcriptomic data.

**Figure 4:**
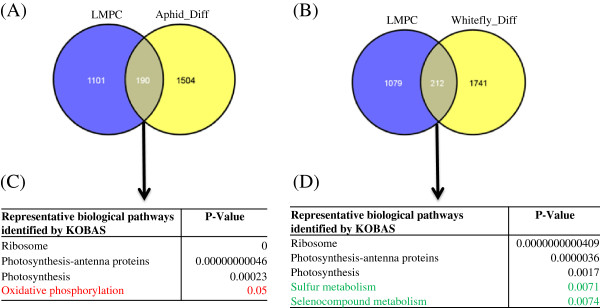
**Venn diagram showing the common and unique genes present in public domain LMPC microarray data (GSE10247) with cotton transcriptomes of aphid-infested (Aphid_diff) (A) and whitefly-infested (Whitefly_diff) (B).** KOBAS analysis of common genes of aphid-infested transcriptome (**C**) and whitefly-infested (**D**) with phloem cell representing genes. Red represents unique pathways in aphids, green represents unique pathways in whitefly genes, and black represents common pathways.

To further evaluate whether cotton and *A. thaliana* share a common pathway in response to infestation by aphids and whiteflies, we compared differentially expressed transcriptomes with those of publically available microarray data sets, namely, GEO: GSE6516 (whitefly induced) [3] and GEO: GSE5525 (aphid induced) [47]. We identified 798 and 134 common differentially expressed transcripts (Figure 3A and 3B) between our transcriptome data and public domain microarray data for aphids and whiteflies, respectively. We further queried 798 and 134 genes against the KOBAS database to understand the common pathways shared between *A. thaliana* and cotton. Our result showed that expression of transcript related to carbohydrate metabolism, ribosome, RNA transport, phagosome, pyruvate, butanoate, and glyoxylate were commonly influnced in both arabidopsis and cotton plant during aphid infestation (Figure 3C). In case of whiteflies, the number of common genes identified for *A. thaliana* and cotton were significantly less (Figure 3B). The common genes in whiteflies exhibit the enrichment of glycerolipid metabolism, ascorbate and aldarate metabolism, glutathion metabolism, ubiquinone-terpenoid quinone biosynthesis, and protein processing in endoplasmic reticulum (Figure 3D) in both *A. thaliana* and cotton. Aphids and whiteflies suck the sap from phloem; so, to evaluate whether there is any differential expression at the transcriptomics level in phloem cells, we compared the differentially expressed transcriptomes of aphid- and whitefly-infested data with publically available phloem transcripts (microarray data sets viz., GEO: GSE10247, Laser microdissected phloem cell-LMPC). We identified 190 and 212 common differentially expressed transcripts (Figure 4A and 4B) between our transcriptome data and the public domain microarray data of the phloem-expressed transcripts for aphids and whiteflies, respectively. We further searched the pathways involved in the phloem cell after infestation by aphids and whiteflies and found that in whiteflies, sulfur metabolism and selenocompound metabolism-related transcripts were differentially expressed; whereas in aphids, oxidative phosphorylation-related transcripts were differentially expressed (Figure 4C and 4D).

### Validation of transcriptomic data using quantitative real-time PCR

**Figure 5:**
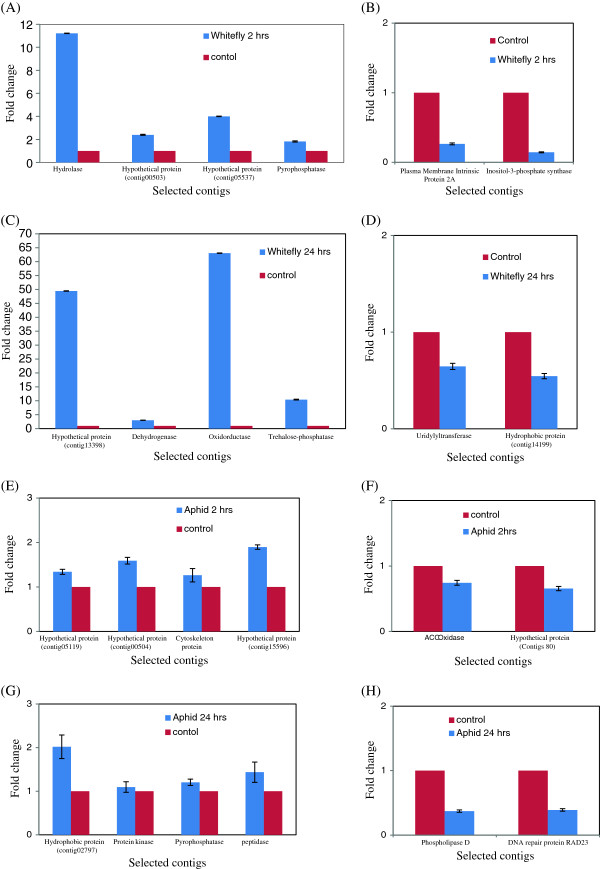
Validation of transcriptome sequencing data by qRT-PCR of selected induced and suppressed contigs, (A and B) Whitefly 2 h; (C and D) Whitefly 24 h; (E and F) Aphid 2 h; and (G and H) Aphid 24 h of infestation.

Six differentially expressed genes including four up-regulated and two down-regulated contigs from each condition as in the case of W2, W24, A2, and A24 were selected on the basis of their differential expression pattern in transcriptomic data for further validation. In case of W2, up-regulated genes like ADP-ribose pyrophosphohydrolase, hypothetical protein (contig00503), hypothetical protein (contig05537), pyrophosphatase and down-regulated genes like plasma membrane intrinsic protein 2A and inositol-3-phosphate synthase were selected. Similalry in case of W24, up-regulated genes like hypothetical protein (contig13398), NADPH dehydrogenase, oxidoreductase, trehalose-phosphatase and down-regulated genes like uridylyltransferase-related and hydrophobic protein (contig14199) were selected. In case of A2, up-regulated genes like hypothetical protein (contig05119), hypothetical protein (contig00504), cytoskeleton protein, hypothetical protein (contig15596) and down-regulated genes like ACC Oxidase and hypothetical protein (contigs 80) were selected. Similalry in case of A24, up-regulated genes like hydrophobic protein (contig02797), protein kinase, H_2_-translocating pyrophosphatase, ATP-dependent peptidase and down-regulated genes like phospholipase D and DNA repair protein RAD23 were selected. The validation of these contigs for each condition, namely, C, W2, W24, A2, and A24, was carried out using three independent biological replicates. All the four induced contigs that were selected for W2 showed an expression that was 2 to 12 fold higher in the whitefly infested condition as compared with their respective control in qRT PCR (Figure 5A). Similarly, in case of W24, the four induced contigs that were selected showed 2 to 65 fold higher expression as compared with non-infested controls (Figure 5C). The down-regulated contigs of W2 and W24 showed their down expression in qRT PCR (Figure 5B and D). Thus, the qRT PCR results on the contigs were selected in complete agreement with the transcriptional data. In case of aphids at both the events, namely, A2 and A24, the four induced contigs that were selected and represented both the events showed higher expression in the aphid-infested condition as compared with the non-infested control; however the range of expression varied from 1.5 to 2 fold (Figure 5E and G). The down-regulated contigs of A2 and A24 showed their down expression in qRT PCR (Figure 5F and H). Further, five contigs viz., 60S ribosomal protein L5, gene representing protein binding, kinase, 60S ribosomal protein L31 and EF-1-alpha were selected for real time validation, which showed constant expression in all the experiments, and this expression was observed as complementing the transcriptomes (Additional file 12). Thus, qRT PCR results agreed with transcriptomic data, however in case of A2 induced genes, the fold on induction over the control was relatively low (Figure 5G).

## Discussion

In the present study, we report the transcriptomic changes in *Gossypium hirsutum* L. leaf, in response to two sap-sucking insects (aphid and whitefly). Cotton plants were infested by these two insects, and transcriptome sequencing at an average 4.4X coverage was completed for the control and infested leaf samples. We observed that plants respond to whiteflies quickly by changing their transcriptome; whereas in case of aphids, the response is slow (Figure 1). The number of down-regulated genes that were more than the up-regulated genes in infestation by both aphids and whiteflies support the previous report which showed that aphids stimulate the suppression of more genes than does induction [47]. Our study suggested that aphids and whiteflies influence the expression of cell and cell wall metabolism by changing the expression of enzymes of sugar metabolism such as phosphoenolpyruvate carboxylase 3, sugar translocator/posphate translocator, cell wall modifier β-Xylosidase 1, inositol oxygenase and cellulose synthase 1(Table 3). We also identified that amino acid metabolism was significantly altered by changing the transcription of key enzymes such as threonin aldolase and 4-hydroxyphenylpyruvate dioxygenase (Table 3). These insects reroute the amino acid transportation [22], and cotton plants probably respond to it by the suppression of the amino acid trans membrane transporter, as a defense strategy that is deployed by plants in response to infestation by aphids. In case of sap-sucking insects, the amino acid composition of plant sap determines the attractiveness of insects [48], and sap-sucking insects, thus, become the secondary sink of amino acid for plants and increase the genes related to the amino acid biosynthesis pathway [10]. It was also reported earlier in *A. thaliana* that *amino acid permease AAP6* mutant reduces the amino acid level in phloem sap and this correlated with the aphid behavior [49]. The infestation mediates the up-regulation of senescence in response to aphids and indicates the breakdown of leaf proteins and probably the translocation of the free amino acid pool, thus forming the phloem sap [31]. The result of cotton transcriptome in response to infestation by aphids and whiteflies showed significantly enrichment of the amino acid biosynthesis pathway (Table 5). During infestation, these insects damage the sieve tube; in response to this damage, plant respond by the release of Ca^2+^, which causes plugging of the sieve plate [28] and prevents the loss of phloem sap. However, aphids overcome this defense by secreting Ca^2+^-binding protein through their saliva, thus preventing clogging. In our experiment, suppression of the Ca^2+^-binding protein was noticed, and this may be considered an insect-influenced plant strategy for increasing the Ca^2+^ level in phloem sap by suppressing these genes (Additional file 9). In qualitative terms, the impact of 2-h infestations by aphids was similar to 24-h infestations by whiteflies; like cytokinin, fungus, bacteria, viruses, JA, gibberellin, and cytokinin responsive transcripts were similarly induced or suppressed by both conditions (Figure 2A and B). Further, we have screened the pathogenic organism that also influenced the expression of these insect (aphid and whitefly) infestation-responsive transcripts with the help of Genvestigator (Additional file 11). Among them, fungi such as *Alternaria brassicicola*, *Botrytis cinerea*, *Blumeria graminis*, *Erysiphe cichoracearum*, *E. orontii*, *Golovinomyces cichoracearum*, *Phytophthora infestans*, and *P. paraistica*; bacteria such as *Escherichia coli* and *Pseudomonas syringae*; and viruses such as cabbage leaf curl virus (CalCUV) and turnip mosaic virus (TuMV) were found. In contrast to chewing insects, weak wounds were created by these phloem feeders. The art-of-style insertion of these insects may be comparable to the fungus haustoria and bacterial infection response. It was reported earlier that intercellular fungal hyphae growth resembles with that of style penetration of whiteflies [50]. The GO annotation of the differentially expressed genes for A2, A24, W2, and W24 showed the involvement of various metabolic and cellular processes (Additional file 8) during infestation by these insects. The transcriptomic reprogramming in response to infestation by aphids and whiteflies showed the up-regulation of several genes belonging to stress, response to signals and pathogens (Additional file 8). Some of the interesting features include the differential expression of transporters in response to sap-sucking insect infestation (Additional file 8); some of transporter-related transcripts are up-regulated in W2, whereas they are down-regulated in A2. The inducibility of water transporter [11], gluatathion S conjugate transporter [22], and sugar transporter [50] was also reported earlier in response to infestation by aphids. In W2, the genes related to various developmental processes such as seed development, post-embryonic development, multicellular development, and root development were found to be induced (Additional file 13). The relationship between developmental genes and biotic stress was reported earlier; for example, seed development genes were found to be induced in response to nematode infestation [51]. The transcripts belonging to secondary metabolic processes such as phenyl propanoid biosynthesis, flavanoids, and aromatic compounds (Additional file 8) were up-regulated during the later phase of infestation by aphids, and these aromatic compounds may be involved in the attraction of parasitoid of aphids [52]. The transcripts of some of the hydrolyses and carboxylesterase were enriched in 2 h of infestation with aphids (Additional file 8). The differential expression of hydrolase and transferase in response to biotic and abiotic stress was shown in the form of the differential expression of glycosyl hydrolase family 1 in *P. rapae* infestation in *Brassica oleracea *[53] and UDP-glycosyltransferase activity in toxic detoxification during insect infestation [54]. The hydrolysis of the product of glucosinolate gives the cyanides and nitriles, which are toxic to herbivores, and the transportation of these to the phloem leads to the deterrence of herbivorous and phloem-feeding insects. The role of glucosinolates in plant defense is well reported; they form in plant tissue and are transported to the site of insect attack [55]. In our result, enzyme benzoate-CoA ligase, which is involved in benzoyloxyglucosinolate synthesis, and genes such as nitrate transporter 1.9 and transporter protein containing the properties of transporters of glucosinolate [56] (Additional file 13) were differentially expressed in response to sap-sucking insect infestation. Similarly, the induction of glutathione S-transferases members in response to cabbage aphids [57], cell wall modification enzymes pectin transferase [58] was reported earlier. The sap-sucking insects are the chief mediators or vectors of spreading plant viruses. There is a decrease in the expression of Poly (A) binding protein 2 (PABP2) in both insect-infested leaves, which help in the transmission of sap-sucking, insect-mediated virus infection and the translation of viral RNA molecule [59], heat shock protein 70 [60], and chloroplast RNA-binding protein 29, which are used in viral protein folding (Additional file 13). Salicylic acid (SA), jasmonic acid (JA), and ethylene signaling pathways are involved in the regulation of plant-induced defense after attacks by pathogens and insects [61]. The JA-responsive pathway is usually activated when there is an attack by necrotrophic and chewing insects; whereas the SA mediates the defense response against biotrophic pathogens and insects such as aphids and whiteflies [62]. Aphids and whiteflies influenced the expression of cotton JA and ET synthesis genes in our experiment. The role of JAR1 in JA-mediated defense development has been already reported. Further, the constitutive expression of JA- and ET-signaling pathways in CEV1 (Constitutive expression of VSP 1) mutant of *Arabidopsis*, which was resistant to aphid growth, was reported [62]. We identified that at a later phase, the infestation of whiteflies leads to the suppression of CEV1 expression (Additional file 4). Thus, our results indicate the fine tuning of the JA pathway in cotton in response to the infestation by aphids and whiteflies. Further, in addition to changes in the expression of the genes involved in JA and ET biosynthesis, we also identified that the expression of hormonal signaling kinases, including MAP2K9 and MAPK6, was also altered, and the relation of MAP kinases in defense mechanisms involving JA, SA, and ET is well established [63]. We also identified that the expression of enzymes involved in oxidative radical scavenging were suppressed after the infestation by aphid and whiteflies (Table 4); these may lead to an increase in oxidative radicals and H_2_O_2_ in the phloem sap, which is a probable strategy that is deployed by cotton plants against insect infestation [38]. We also report the involvement of ABA and GA pathways during the infestation by aphids and whiteflies in cotton (Figure 2A). The role of ABA [64,65] and GA [66] in plant–insect interaction has been recently shown. Further, the involvement of ABA and GA during the defense responses against green bug phloem feeding in sorghum [67] has been recently demonstrated. It has been reported that the increase in GA causes the increase in trichome density, and this may protect the plants from aphids and whiteflies [66]. Our results further showed the involvement of cytokinin in defense responses to aphids and especially whiteflies (Figure 2A). The involvement of cytokinins in defense responses toward aphids or whiteflies has not been reported earlier; however, the role of cytokinins in plants and bacterial interactions [68] and bacterial isopentenyl transferase (ipt) genes, which are involved in cytokinin biosynthesis, which, in turn, are involved in resistance to the tobacco hornworm and green peach aphid nymphs, was reported [69]. The down-regulation of genes such as overexpressors of cationic peroxidase 3- OCP3 (Additional file 9 and Additional file 10B), non-specific lipase [45], LOX1 [70], and TGA2 [71] which leads to the suppression of phytohormonal-mediated plant resistance and increase in the expression of DMR6 (Additional file 4 and Additional file 10A), which is a positive regulator of the susceptibility of plants to pathogens [46], showed insect-mediated suppression of plant defense and compatible infestation of these insects. Plants assimilate heavy metals such as Ni [72], Zn [73], and Se [74] for protection against herbivorous insects. We identified the enrichment of selenometallo metabolism in the case of whiteflies infested both cotton and *Arabidopsis* plant (Figure 4D). Selenium is a member of sulfur(s) group, and, hence, plants readily assimilate selenate in place of sulfur into cysteine as selenocysteine (SeCys) via the sulfur metabolic pathway [74]; this explains the enrichment of the sellanometallo metabolic pathway in response to whiteflies. We also observed the enrichment of the transcript related to RNA transport both in cotton and *Arabidopsis* plants during aphid infestation (Figure 3C). It is already reported that viruses hijack the plant RNA transportation system for disease spreading. In parellal, plant activate the pathway of mRNA surveillance to control formation of aberrant RNA, which is a defense mechanism, was also generated in response to virus infection in plants [75]. Sap-sucking insects are potential vectors for plant-borne viruses [10]; thus, our result also proposes an interesting question as to whether plants understand the potential threat of virus infection after the infestation by aphids and whiteflies.

## Conclusions

An average of 4X coverage transcriptome information will be helpful in understanding the induced defense responses operating against aphids and whiteflies in agriculturally important cotton plants, and will also pave the way for developing new insect pest-management strategies. The expression pattern of transcripts reveals that sap-sucking insects interact with plants by suppressing the expression of positive regulators of phytohormonal-induced resistance genes, inducing the negative regulator of the plant resistance gene and suppressing the defense-related transcription factors such as WRKY and other MAP kinases involved in plant defense. Our result also suggests that these insects shift the sucrose and amino acid mobilization by changing the expression pattern of different genes related to amino acid and carbohydrate metabolism. Thus, insects facilitate their infestations and plants try to repel these insects by activating their glucosinolates, secondary metabolite pathway, and reactive oxygen weapons.

## Methods

### Plant material and insect infestation

The seeds of *Gossypium hirsutum* (var.MCU5) were sown for germination in a mixture of solarite, vermiculite, garden soil, and sand soil (1:1:1:1 ratio). After germination of seeds, plants were grown for five weeks in a glass house at 28 ± 2°C (day/night), a relative humidity of 50–60%, a 14-h photoperiod, and a photosynthetically active radiation of 900 μmol m-^2^ s-^1^, respectively. Five-week-old plants containing six leaves were selected for the experiments. Cultures of aphid and whiteflies were maintained in potted cotton plants in the laboratory at 26 ± 2°C and 70% relative humidity [76]. About 25 freshly emerged whiteflies and 2nd instars aphid nymphs were released an average per leaf of plants. Whole experimental plants were covered with perforated polyethylene bags to prevent the insects from escaping. The insect infestation experiments were performed in three biological replicates. Solutions of 10 mM MgCl_2_ (pH 7.0 /dissolve in PBS) were spread as mock solution [77]. Insects were removed by a fine brush after 2 and 24 h of infestation, and immediately, two middle leaves were frozen in liquid N_2_ for total RNA isolation. All the experiments with Aphids and whiteflies were performed with approval of IBSC(Institutional Biosafety Committee).

### RNA isolation and sample preparation

Total RNA were isolated by using Spectrum plant total RNA Kit (Sigma-Aldrich) according to the manufacturer’s protocol and underwent DNaseI treatment. Out of three biological replicates, only one plant’s RNA was used for transcriptome sequencing. To amplify the mRNA, double-stranded cDNA were prepared using oligo-dT primers containing T7 promoter and SuperScript® Double-Stranded cDNA Synthesis Kit (Invitrogen). These double-stranded cDNA were amplified using an in vitro amplification system of the Gene chip IVT labeling kit (Affymetrix). Amplified cRNA underwent double-stranded cDNA preparation by using random hexamer primer and SuperScript® Double-Stranded cDNA Synthesis Kit (Invitrogen). These double-stranded cDNA were purified by the QIAquick PCR purification column (Qiagen). The double-stranded cDNA (3 μg) were used for transcriptome sequencing. The transcriptome sequencing was performed as per the manufacturer’s protocol of Roche’s GS-Titanium pyrosequencing. Further to confirm linearity in expression pattern among the three biological replicate, microarray experiments were performed with isolated RNA sample through Affymetrix kit as per the manufacturer’s protocol.

### Assembly and annotation of transcriptomes

The reads from each library were assembled separately by Roche Newbler (GS-assembler) version 2.3. The assembly criteria were set as 40 bp overlap size and 90% identity between the reads. Contigs of each library were pooled together and assembled to form a common data set. The reads were counted from their respective libraries for the newly assembled contigs, and their TPM (Transcript per Million) was calculated. For further analysis, TPM values were log2 transformed (Additional file 14). Genes such as Actin (AY305733), UBQ7 (DQ116441), Gbpolyubiquitin-1 (AY375335), Gbpolyubiquitin-2 (EE592464), Histone 3 (AF024716), and 18S rRNA (L24145) [78] were selected for the normalization of the expressed contigs in each condition. Digital gene Expression was carried out by DEGseq package in R-bioconductor 2.15 for each library with reference to control. Contigs with p-value ≥ 0.05 were selected for differential gene expression. Two-fold up- and two-fold down-regulated contigs were selected. Therefore, 8 categories were made (control vs. aphid 2 h up, control vs. aphid 2 h down, control vs. aphid 24 h up, control vs. aphid 24 h down, control vs. whitefly 2 h up, control vs. whitefly 2 h down, control vs. whitefly 24 h up, and control vs. whitefly 24 h down) (Additional file 4). The differential genes for each transcripts were subjected to 2 by 2 Chi-square test. The test was performed between the differential genes of A2 vs W2 and A24 vs W24. The p-value for A2 and W2 was found to be 0.002 showing the 99% significance level while in case of A24 and W24 we got the p-value of 0.809 only. The contigs of each event were subjected to blast using program blastx with the TAIR 9 protein database (Additional file 15) and blastn for cotton ESTs available in the NCBI database (Additional file 16) at e-value 10^-5^.

### Functional annotation

The TAIR IDs of the contigs in each event were used for the GO annotation. The detailed GO annotations were studied using the agriGO tool (http://bioinfo.cau.edu.cn/agriGO/), which was categorized in biological processes, molecular functions. The differential genes were querid against the hormonal and biotic stress related transcripts in genevestigator (https://www.genevestigator.com/gv/plant.jsp). All the differentially expressed genes were also subjected to KOBAS analysis (http://kobas.cbi.pku.edu.cn/home.do), and significant pathways were selected at the p value ≤ 0.05. Differentially expressed genes were also compared with the public databases generated from plants of *Arabidopsis thaliana* that were infested with aphids and whiteflies at different time points (GEO: GSE5525, GEO: GSE6516) and Laser Microdetection Phloem Cells (LMPC - GEO: GSE10247), which were derivatives of *Arabidopsis thaliana*. The genes that were common in both data sets were studied, and the significant pathways were retrieved at p value ≤ 0.05 by using KOBAS.

### Real-time PCR analysis

Real-time PCR analysis was performed in biological triplicates. DNase I-treated RNA (2μg) were converted into cDNA using SuperScript® III First-Strand Synthesis kit (Invitrogen). The cDNA products were diluted 10 fold with deionized water before use as a template in real-time PCR. The quantitative reaction was performed on an ABI 7500 Real-Time PCR Detection System (Applied Biosystems) using the SYBR Green PCR Master Mix (Applied Biosystems, CA, USA). The reaction mixture (20 μL) contained 2X SYBR Green PCR Master mix, 1μl (10 pmol) each of the forward and reverse primers, and 1 μL of diluted cDNA. PCR amplification was performed under the following conditions: 95°C for 20s, followed by 40 cycles of 95°C for 3s and 62°C for 30s. The expressions of selected contigs were normalized against an internal reference gene ubiquitin (AY375335F). The relative gene expression was calculated using the 2^-ΔΔCt^ method [79]. All primers used in this study are listed in Additional file 17.

## Abbreviations

cDNA: Complementary deoxyribonucleic acid; IVT: *In vitro*-transcription; GO: Gene ontology; EST: Expressed sequence tag; TPM: Transcript per million; SA: Salicylic acid; JA: Jasmonic acid; ET: Ethylene; A2: Aphid 2 h of infestation; A24: Aphid 24 h of infestation; W2: Whitefly 2 h of infestation; W24: Whitefly 24 h of infestation; F: Molecular function; P: Biological process.

## Competing interests

The authors declare that they have no competing interests.

## Authors’ contributions

NKD performed transcriptome sequencing, real time PCR, participate in data analysis and drafted the manuscript. RG and SKB helped in computational and statistical analysis of transcriptomic data. AR, AI and SKS helped in lab experiments. KC helped in insect infestation experiment. KDP and PKS revised the manuscript. SVS conceived the study and participated in its design and coordination. All authors read and approved the final manuscript.

## Supplementary Material

Additional file 1 **Contigs size distribution.** JEPG file showed contigs size distribution of assembled reads obtained from transcriptome sequencing at different time points of infestation with aphids and whiteflies. Click here for file

Additional file 2 **Singletons size distribution.** JEPG file showed size distribution of singletons obtained at different time points of infestation by aphids and whiteflies. Click here for file

Additional file 3 **Size distributions (in base pair) of generated contigs of common data set.** JEPG file showed size distribution of generated contigs of common data set. Click here for file

Additional file 4 **Differentially up- and down-regulated contigs (≥ 2 fold) expression in TPM in comparison to the control.** Excel file containing the list of contigs that are more than 2 fold up- and down-regulated in different sets of aphid- and whitefly-infested experiments. Click here for file

Additional file 5 **Average fold change of aphid and whitefly infestation mediated differentially expressed transcript.** Excel file containing the average fold-change expression of cotton transcriptomes after infestation by aphids and whiteflies. Click here for file

Additional file 6 **Expression pattern of biological replicate in microarray experiment for Control condition.** Figure of expression pattern of biological triplicate in microarray experiment for Control Condition. Third sample was selected for transcriptome sequencing. Click here for file

Additional file 7 **Expression pattern of biological replicate in microarray experiment for Whitefly infestation.** Figure of expression pattern of biological triplicate in microarray experiment for Whitefly infestation. Third sample was selected for transcriptome sequencing. Click here for file

Additional file 8 (**A**) **GO annotation of ≥2 fold up- and down-regulated genes represented in molecular functions (F) and biological processes (P).** C-A2_up and C-A24_up represent aphid 2 and 24 h infestation’s up-regulated genes; whereas C-A2_down and C-A24_down represent aphid infestation’s down-regulated gene as compared with the control. (**B**) GO annotation of ≥2 fold up- and down-regulated genes represented in molecular functions (F) and biological processes (P). C-W2_up and C-W24_up represent whitefly 2 and 24 h infestation’s up-regulated genes; whereas C-W2_down and C-W24_down represent whitefly infestation’s down-regulated gene as compared with the control. Click here for file

Additional file 9 **List of induced and suppressed defense-related genes.** Excel file containing the induced as well as defense-related transcripts and their fold-change expression in experiments depicting infestation by aphids and whiteflies. Click here for file

Additional file 10 **Expression pattern of overexpressors of cationic peroxidase 3 and downy mildew resistance 6 gene in response to infestation by aphids and whiteflies with RT-PCR.** Figure shows Downy mildew resistance 6 gene (At5g24530) (A) and cationic peroxidase 3 (At5g11270) (B) in response to infestation by aphids and whiteflies with real-time PCR. Click here for file

Additional file 11 **Different pathogens that influence the expression pattern of aphid- and whitefly induced genes.** JEPG file showing pathogens that also influence the expression of genes which showed differential expression after infestation by aphids and whiteflies in cotton. Click here for file

Additional file 12 **Expression pattern by qRT-PCR of selected constant expressive contigs.** Validation of transcriptome sequencing data by qRT-PCR of selected contigs that have constant expression throughout the experiment. Click here for file

Additional file 13 **Expression profile of developmental, virus multiplication and glucosinolate transporter- related transcripts in TPM during different stages of insect infestation.** Excel file containing the comparative TPM of developmental and virus multiplication-related transcripts during infestation by aphids and whiteflies. Click here for file

Additional file 14 Comparative transcript per million (TPM) of generated contigs. Excel file containing the comparative TPM analysis of generated contigs. Click here for file

Additional file 15 TAIR9 Protein Database BLAST Result. Excel file containing the annotation of contigs of common data set with the TAIR9 protein database by the blastx program at the e-value 10-5. Click here for file

Additional file 16 Cotton EST BLAST Result. Excel file containing the annotation of contigs of common data set with the cotton EST gene list by the blastn program at the e-value 10-5. Click here for file

Additional file 17 **Primer sequences of selected contigs used in qRT-PCR.** Excel file containing the selected contigs and their primer sequences used in validation of transcriptomes. Click here for file
