# Supplementary figures and images for: Comparative transcriptome analysis of Gossypium hirsutum L. in response to sap sucking insects: aphid and whitefly
Source: BMC Genomics. 2013 Apr 11;14:241. doi: 10.1186/1471-2164-14-241 (PMC3637549; doi:10.1186/1471-2164-14-241)

# Additional File 1

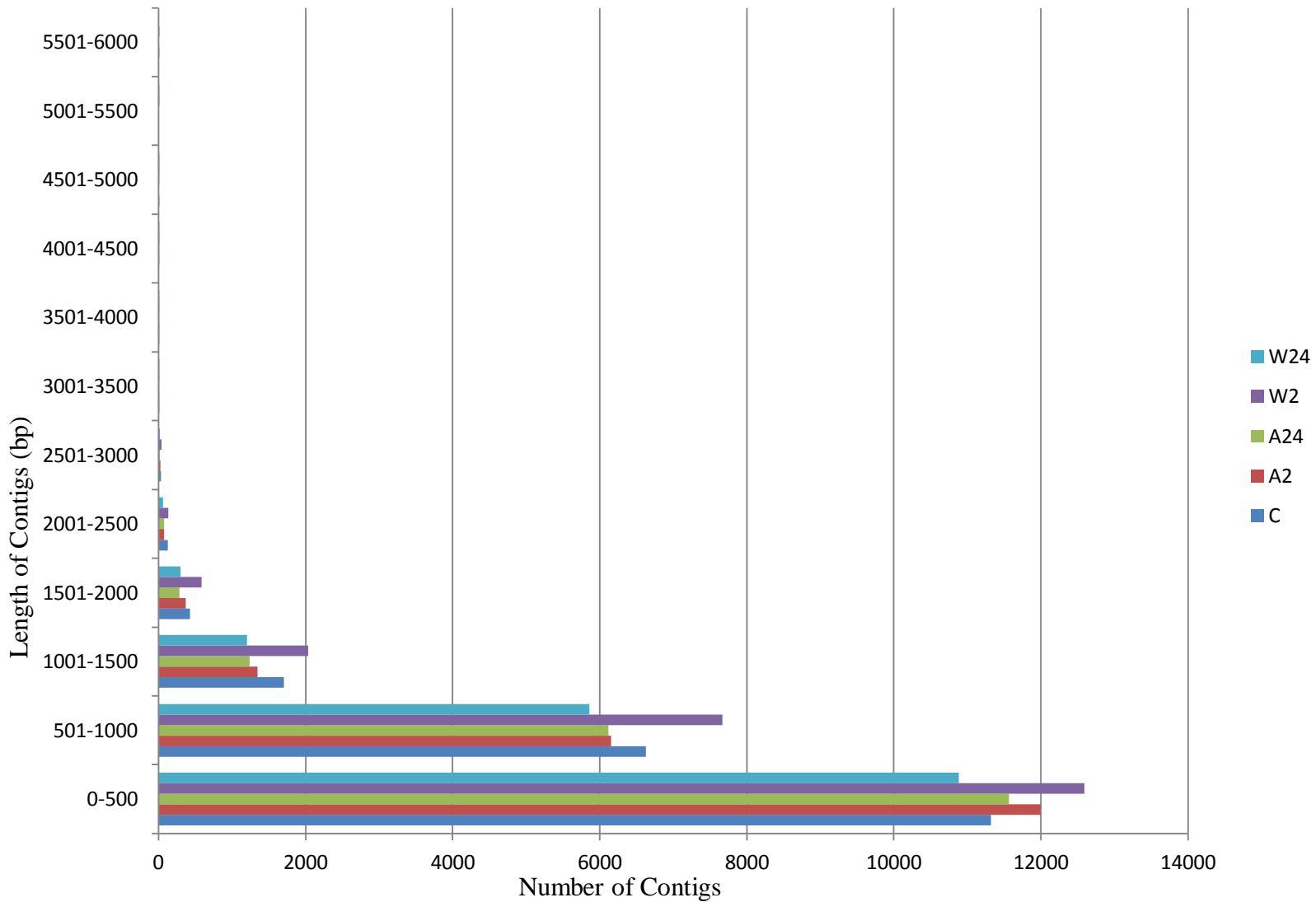

Supplement: Additional file 1 — Contigs size distribution. JEPG file showed contigs size distribution of assembled reads obtained from transcriptome sequencing at different time points of infestation with aphids and whiteflies. [file 1471-2164-14-241-S1.pdf]

## Additional File 2

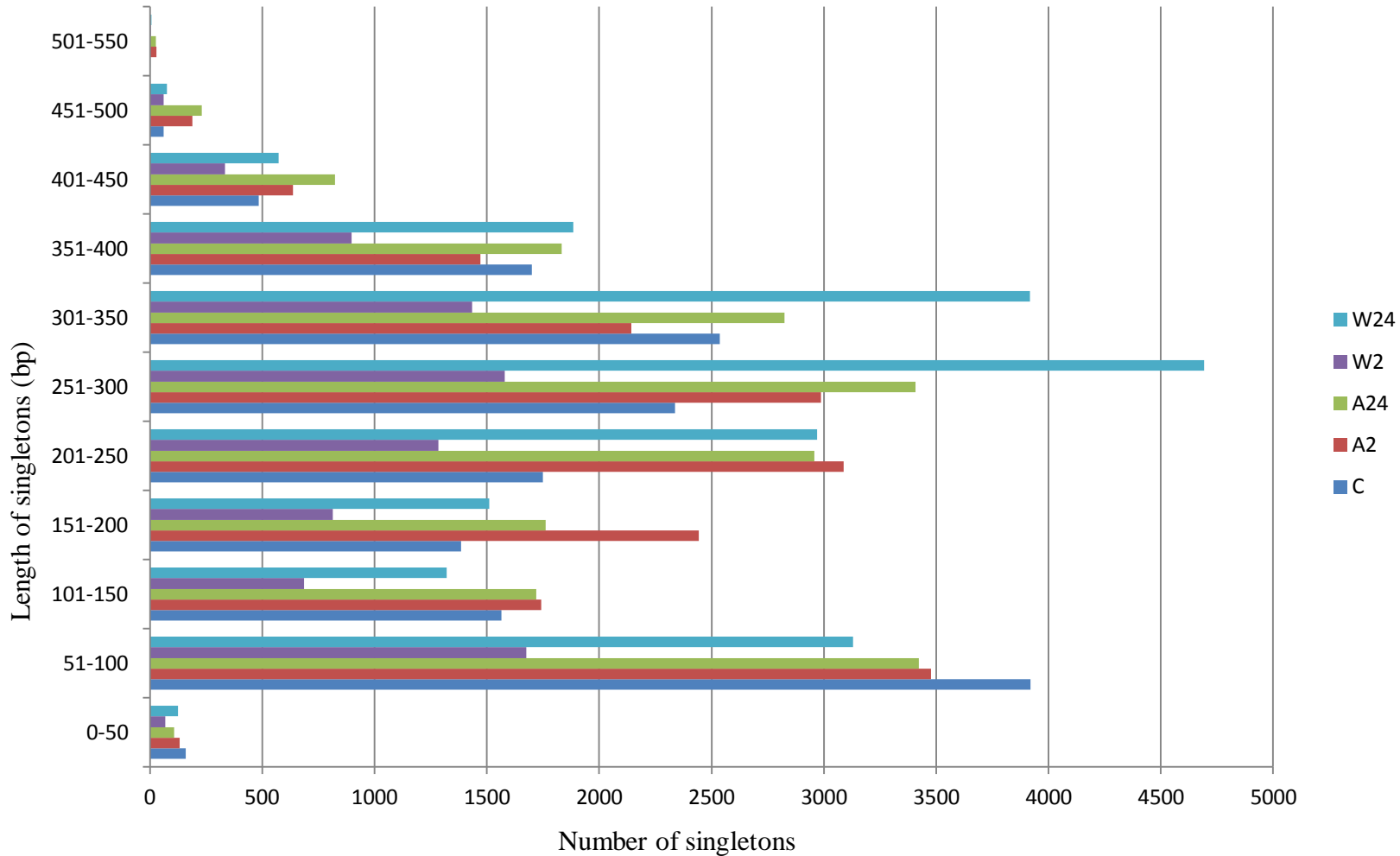

Singletons size distribution.

Supplement: Additional file 2 — Singletons size distribution. JEPG file showed size distribution of singletons obtained at different time points of infestation by aphids and whiteflies. [file 1471-2164-14-241-S2.pdf]

## Additional File 3

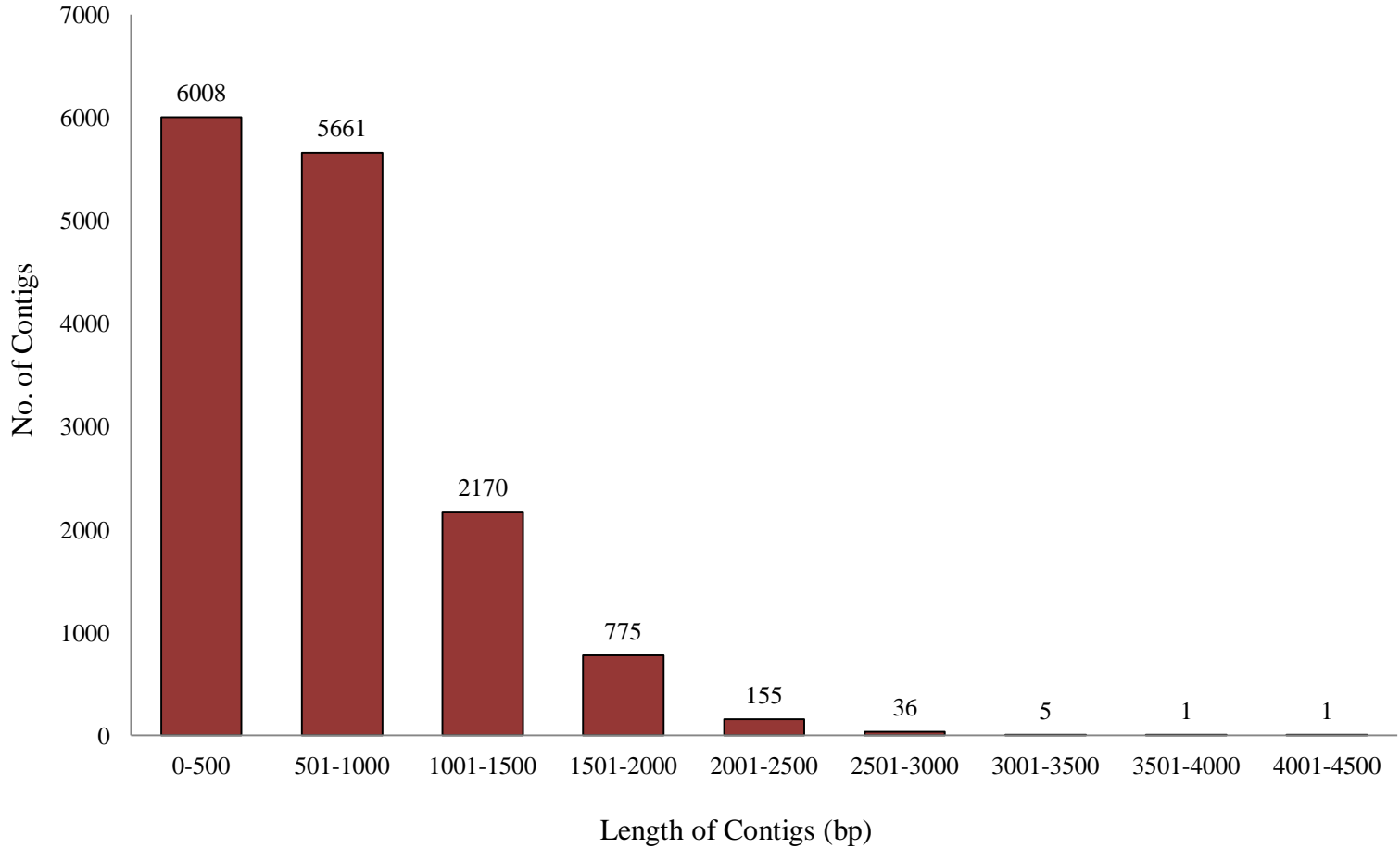

Size distributions (in base pair) of generated contigs of common data set.

Supplement: Additional file 3 — Size distributions (in base pair) of generated contigs of common data set. JEPG file showed size distribution of generated contigs of common data set. [file 1471-2164-14-241-S3.pdf]
