# Supplementary material for: Comparative transcriptome analysis of Gossypium hirsutum L. in response to sap sucking insects: aphid and whitefly
Source: BMC Genomics. 2013 Apr 11;14:241. doi: 10.1186/1471-2164-14-241 (PMC3637549; doi:10.1186/1471-2164-14-241)
Supplement: Additional file 6 — Expression pattern of biological replicate in microarray experiment for Control condition. Figure of expression pattern of biological triplicate in microarray experiment for Control Condition. Third sample was selected for transcriptome sequencing. [file 1471-2164-14-241-S6.pdf]

Additional file 6

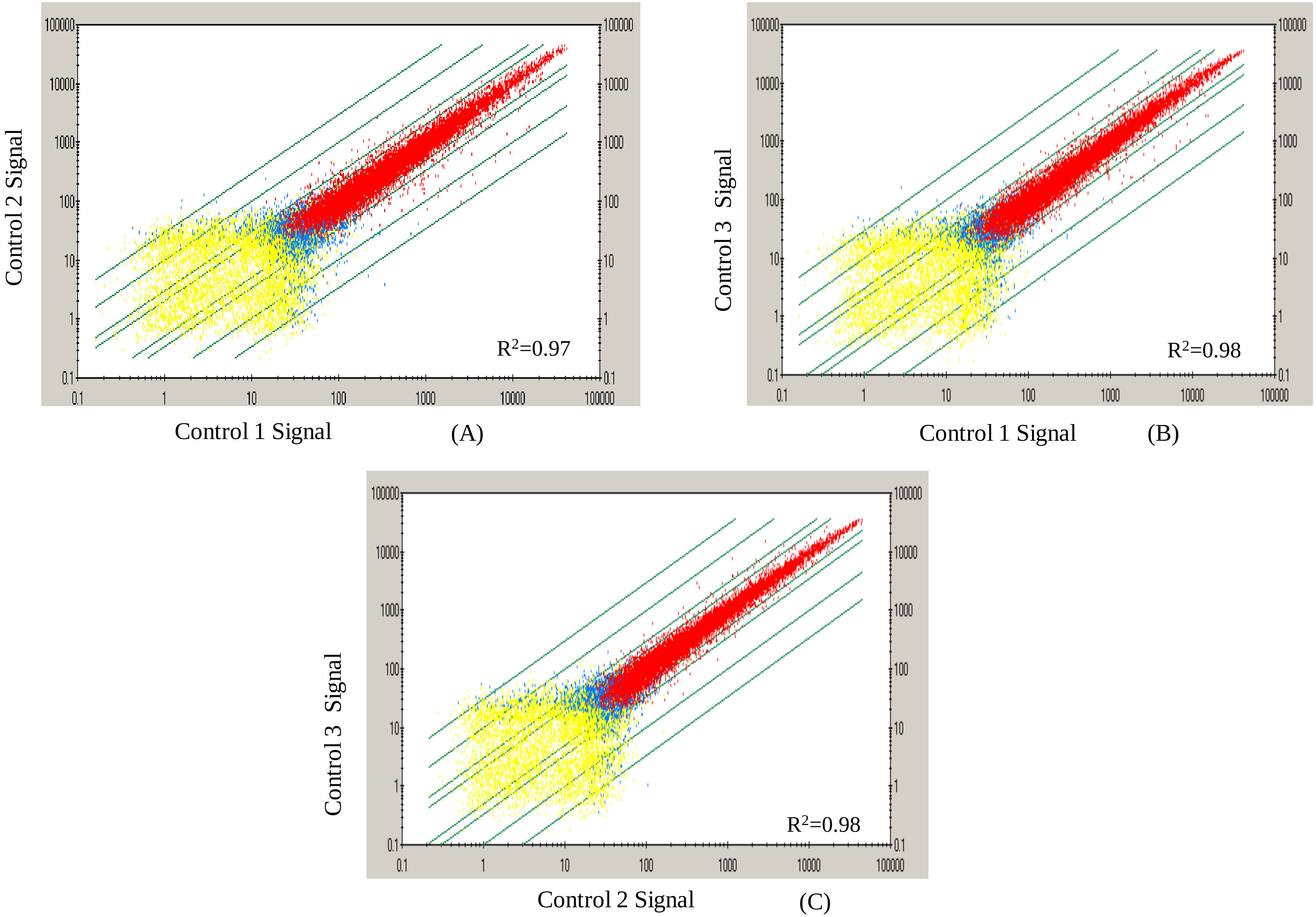

Expression pattern of biological replicate in microarray experiment for Control Condition.
