# Supplementary material for: Comparative transcriptome analysis of Gossypium hirsutum L. in response to sap sucking insects: aphid and whitefly
Source: BMC Genomics. 2013 Apr 11;14:241. doi: 10.1186/1471-2164-14-241 (PMC3637549; doi:10.1186/1471-2164-14-241)
Supplement: Additional file 8 — (A) GO annotation of ≥2 fold up- and down-regulated genes represented in molecular functions (F) and biological processes (P). C-A2_up and C-A24_up represent aphid 2 and 24 h infestation’s up-regulated genes; whereas C-A2_down and C-A24_down represent aphid infestation’s down-regulated gene as compared with the control. (B) GO annotation of ≥2 fold up- and down-regulated genes represented in molecular functions (F) and biological processes (P). C-W2_up and C-W24_up represent whitefly 2 and 24 h infestation’s up-regulated genes; whereas C-W2_down and C-W24_down represent whitefly infestation’s down-regulated gene as compared with the control. [file 1471-2164-14-241-S8.pdf]

Additional file 8

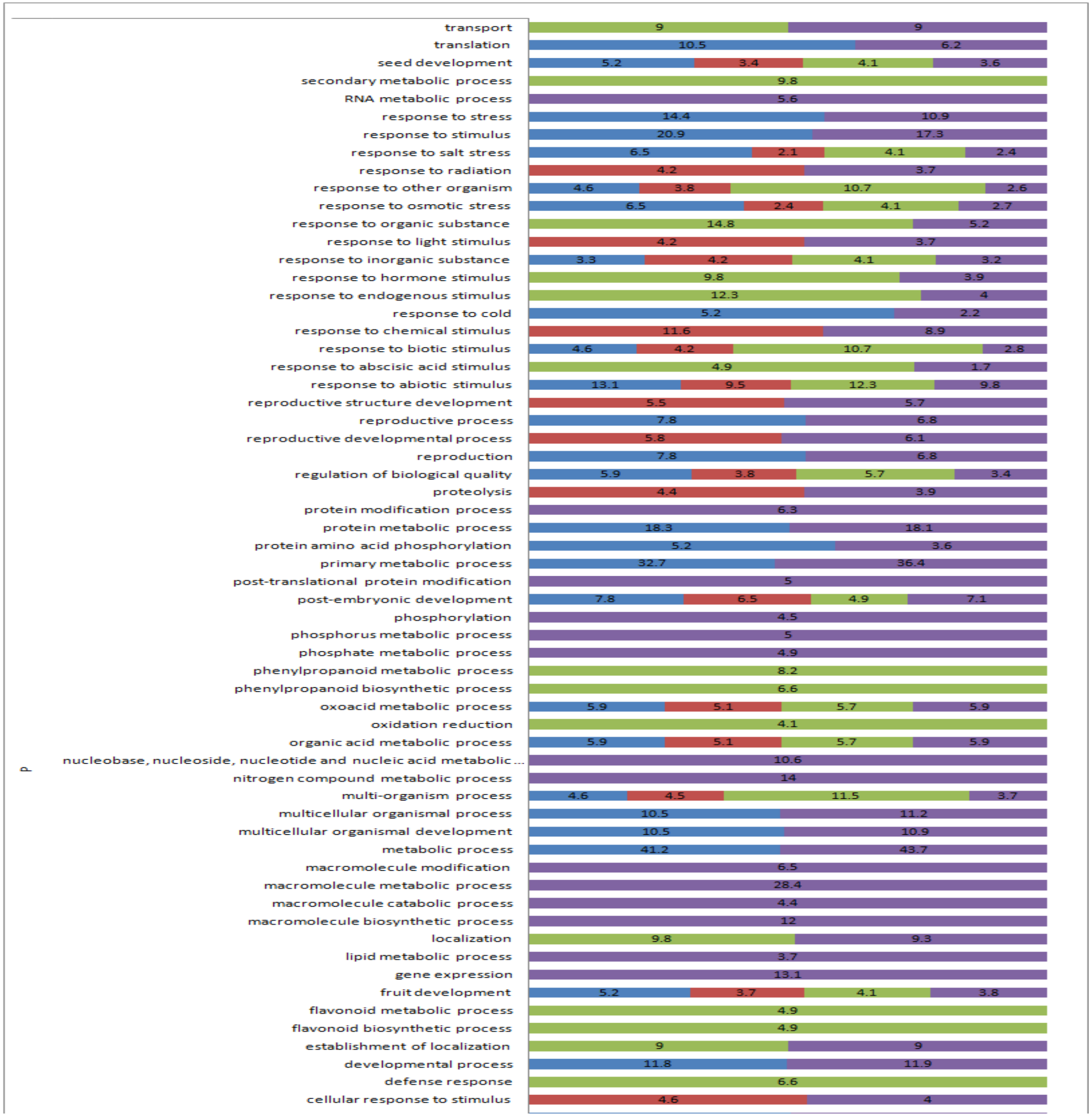

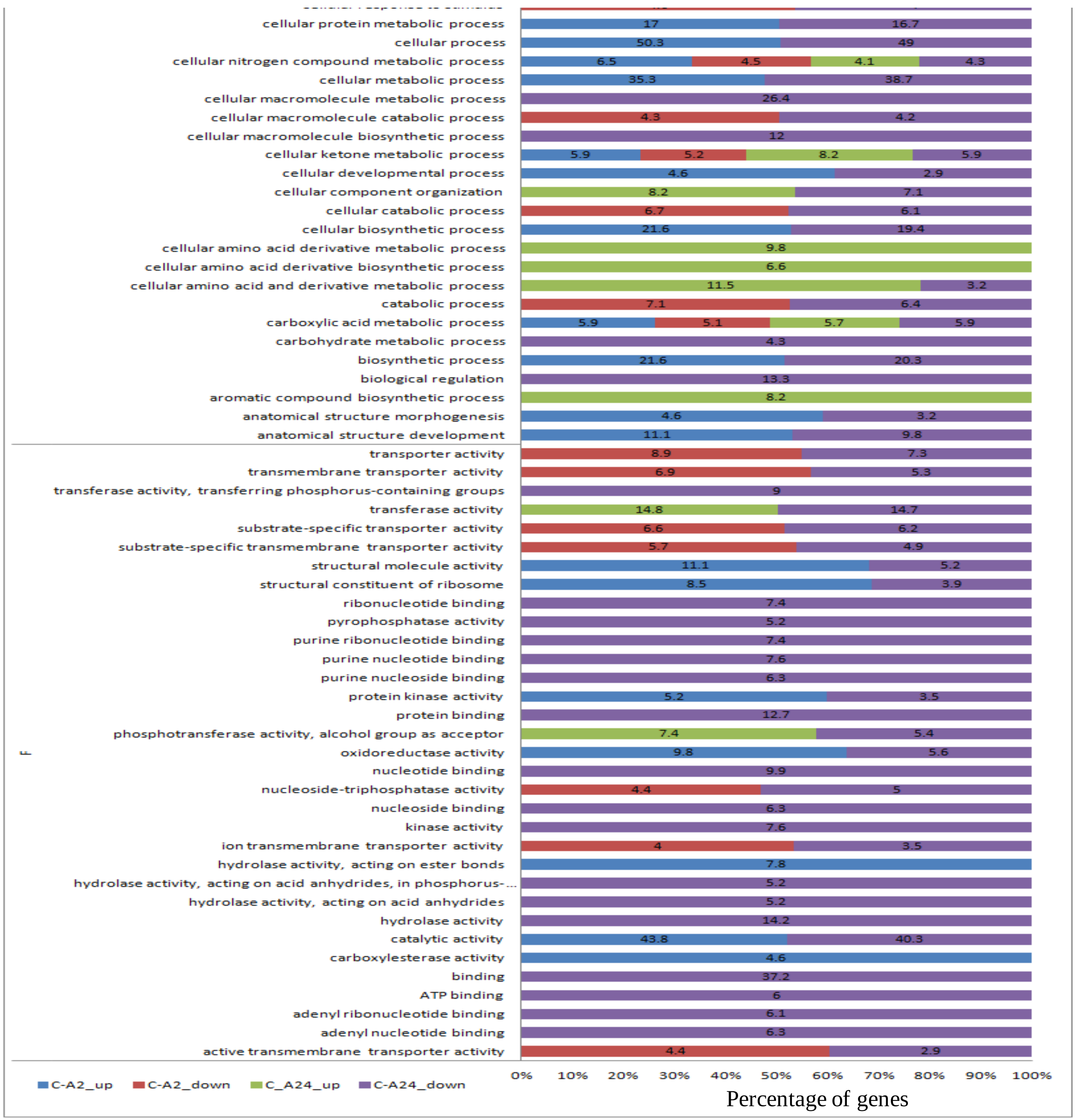

A GO annotation of ≥2 fold up- and down-regulated genes represented in molecular functions (F) and biological processes (P). C-A2_up and C-A24_up represent aphid 2 and 24 h infestation's up-regulated genes; whereas C-A2_down and C- A24_down represent aphid infestation's down-regulated gene as compared with the control.

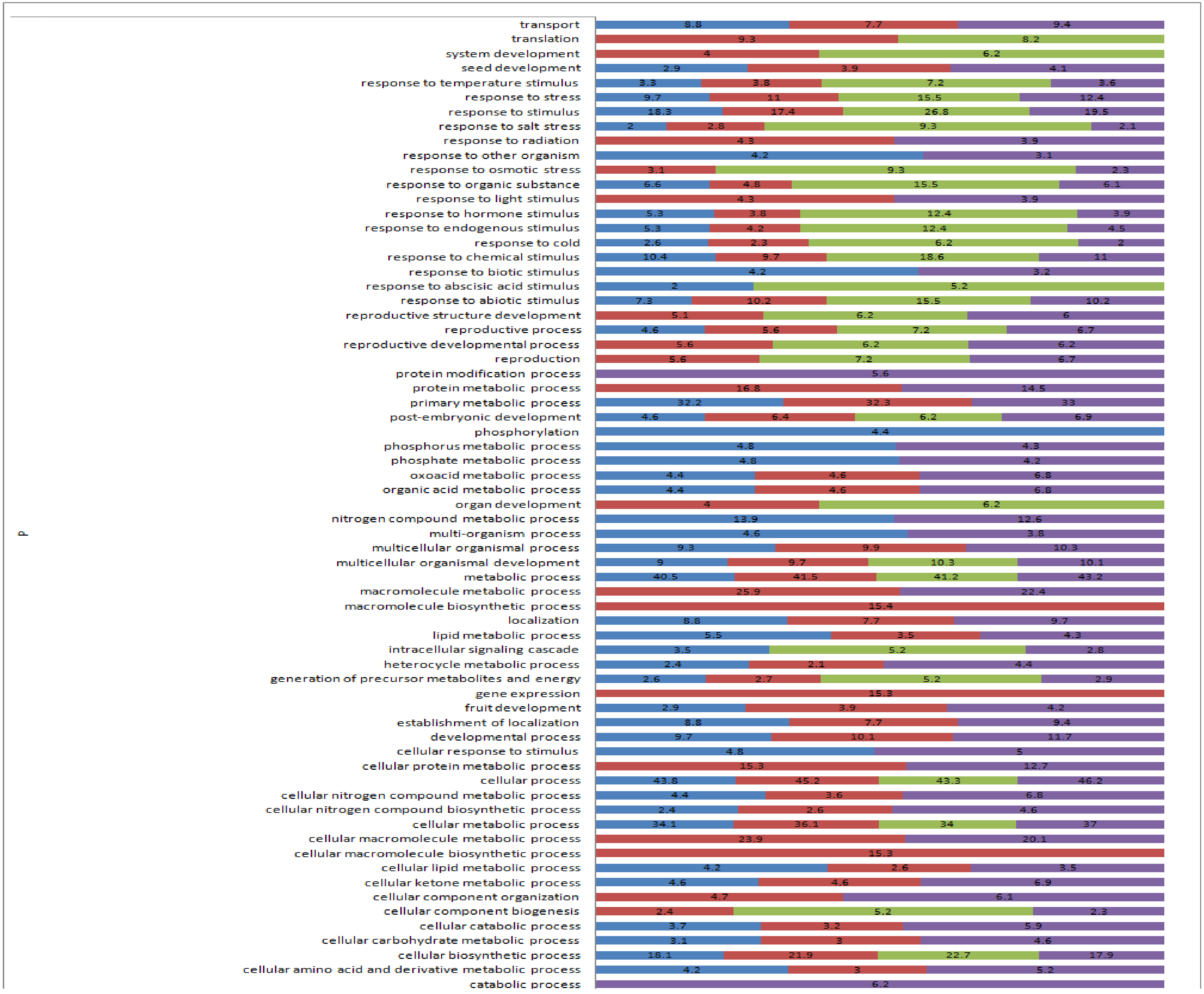
P
transport 8.8 7.7 9.4
translation 9.3 8.2
system development 4 6.2
seed development 2.9 3.9 4.1
response to temperature stimulus 3.3 3.8 7.2 3.6
response to stress 9.7 11 15.5 12.4
response to stimulus 18.3 17.4 26.8 19.5
response to salt stress 2 2.8 9.3 2.1
response to radiation 4.3 3.9
response to other organism 4.2 3.1
response to osmotic stress 3.1 9.3 2.3
response to organic substance 6.6 4.8 15.5 6.1
response to light stimulus 4.3 3.9
response to hormone stimulus 5.3 3.8 12.4 3.9
response to endogenous stimulus 5.3 4.2 12.4 4.5
response to cold 2.6 2.3 6.2 2
response to chemical stimulus 10.4 9.7 18.6 11
response to biotic stimulus 4.2 3.2
response to abscisic acid stimulus 2 5.2
response to abiotic stimulus 7.3 10.2 15.5 10.2
reproductive structure development 5.1 6.2 6
reproductive process 4.6 5.6 7.2 6.7
reproductive developmental process 5.6 6.2 6.2
reproduction 5.6 7.2 6.7
protein modification process 5.6
protein metabolic process 16.8 14.5
primary metabolic process 32.2 32.3 33
post-embryonic development 4.6 6.4 6.2 6.9
phosphorylation 4.4
phosphorus metabolic process 4.8 4.3
phosphate metabolic process 4.8 4.2
oxoacid metabolic process 4.4 4.6 6.8
organic acid metabolic process 4.4 4.6 6.8
organ development 4 6.2
nitrogen compound metabolic process 13.9 12.6
multi-organism process 4.6 3.8
multicellular organismal process 9.3 9.9 10.3
multicellular organismal development 9 9.7 10.3 10.1
metabolic process 40.5 41.5 41.2 43.2
macromolecule metabolic process 25.9 22.4
macromolecule biosynthetic process 15.4
localization 8.8 7.7 9.7
lipid metabolic process 5.5 3.5 4.3
intracellular signaling cascade 3.5 5.2 2.8
heterocycle metabolic process 2.4 2.1 4.4
generation of precursor metabolites and energy 2.6 2.7 5.2 2.9
gene expression 15.3
fruit development 2.9 3.9 4.2
establishment of localization 8.8 7.7 9.4
developmental process 9.7 10.1 11.7
cellular response to stimulus 4.8 5
cellular protein metabolic process 15.3 12.7
cellular process 43.8 45.2 43.3 46.2
cellular nitrogen compound metabolic process 4.4 3.6 6.8
cellular nitrogen compound biosynthetic process 2.4 2.6 4.6
cellular metabolic process 34.1 36.1 34 37
cellular macromolecule metabolic process 23.9 20.1
cellular macromolecule biosynthetic process 15.3
cellular lipid metabolic process 4.2 2.6 3.5
cellular ketone metabolic process 4.6 4.6 6.9
cellular component organization 4.7 6.1
cellular component biogenesis 2.4 5.2 2.3
cellular catabolic process 3.7 3.2 5.9
cellular carbohydrate metabolic process 3.1 3 4.6
cellular biosynthetic process 18.1 21.9 22.7 17.9
cellular amino acid and derivative metabolic process 4.2 3 5.2
catabolic process 6.2

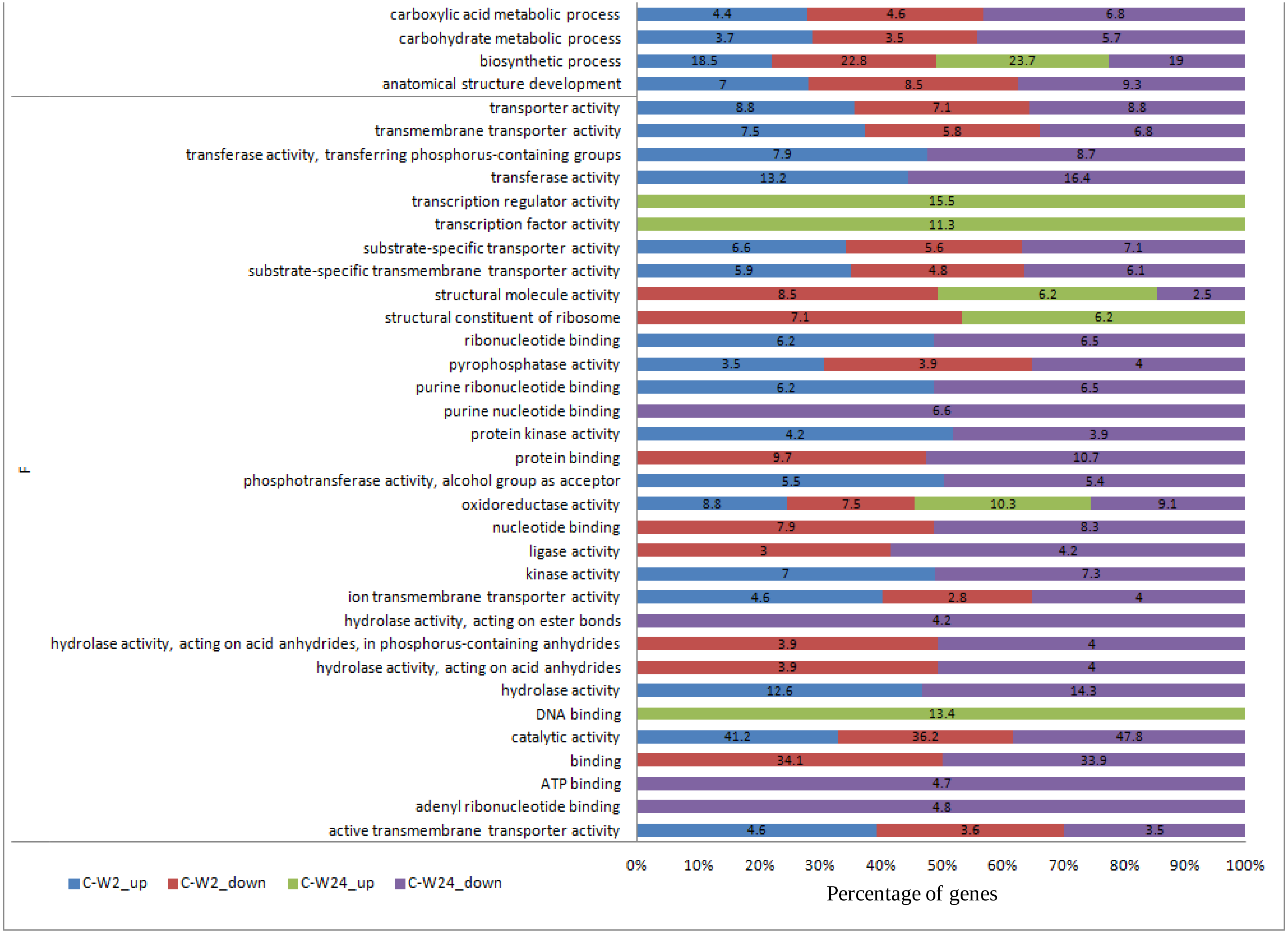

B GO annotation of ≥2 fold up- and down-regulated genes represented in molecular functions (F) and biological processes (P). C-W2_up and C-W24_up represent whitefly 2 and 24 h infestation's up-regulated genes; whereas C-W2_down and C-W24_down represent whitefly infestation's down-regulated gene as compared with the control.
