# Supplementary material for: Comparative transcriptome analysis of Gossypium hirsutum L. in response to sap sucking insects: aphid and whitefly
Source: BMC Genomics. 2013 Apr 11;14:241. doi: 10.1186/1471-2164-14-241 (PMC3637549; doi:10.1186/1471-2164-14-241)
Supplement: Additional file 10 — Expression pattern of overexpressors of cationic peroxidase 3 and downy mildew resistance 6 gene in response to infestation by aphids and whiteflies with RT-PCR. Figure shows Downy mildew resistance 6 gene (At5g24530) (A) and cationic peroxidase 3 (At5g11270) (B) in response to infestation by aphids and whiteflies with real-time PCR. [file 1471-2164-14-241-S10.pdf]

Additional file 10

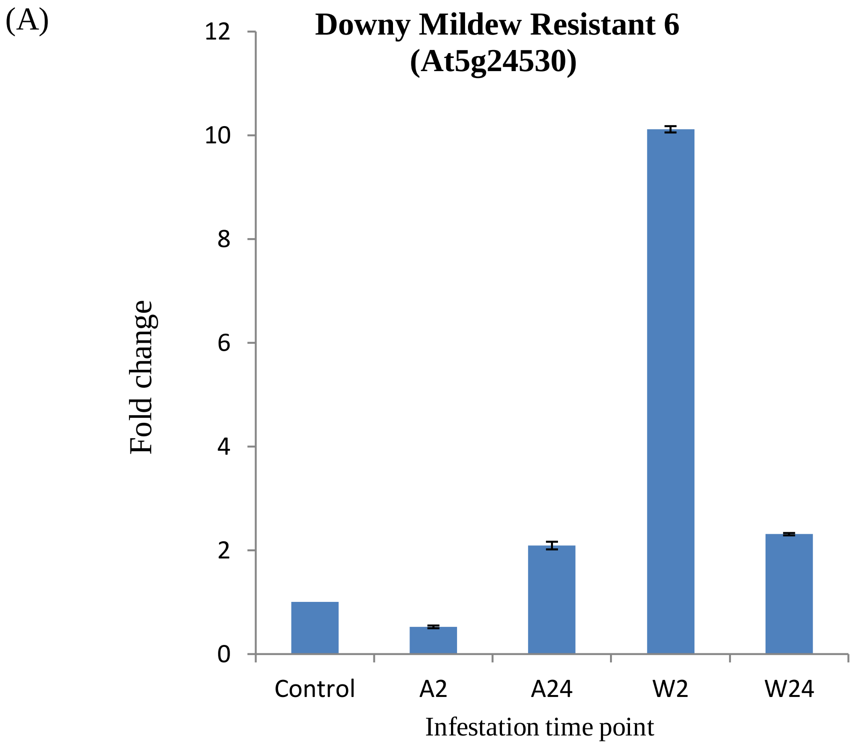

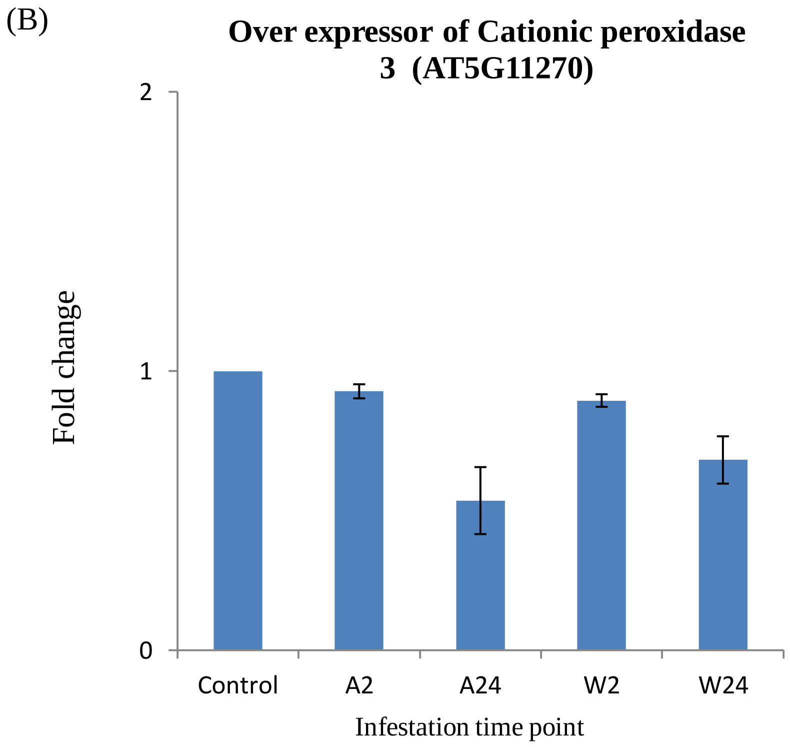

Expression pattern of overexpressors of downy mildew resistance 6 gene (At5g24530)(A) and cationic peroxidase 3 (At5g11270) (B) in response to infestation by aphids and whiteflies with real-time PCR.
