# Supplementary material for: Comparative transcriptome analysis of Gossypium hirsutum L. in response to sap sucking insects: aphid and whitefly
Source: BMC Genomics. 2013 Apr 11;14:241. doi: 10.1186/1471-2164-14-241 (PMC3637549; doi:10.1186/1471-2164-14-241)
Supplement: Additional file 11 — Different pathogens that influence the expression pattern of aphid- and whitefly induced genes. JEPG file showing pathogens that also influence the expression of genes which showed differential expression after infestation by aphids and whiteflies in cotton. [file 1471-2164-14-241-S11.pdf]

Additional file 11

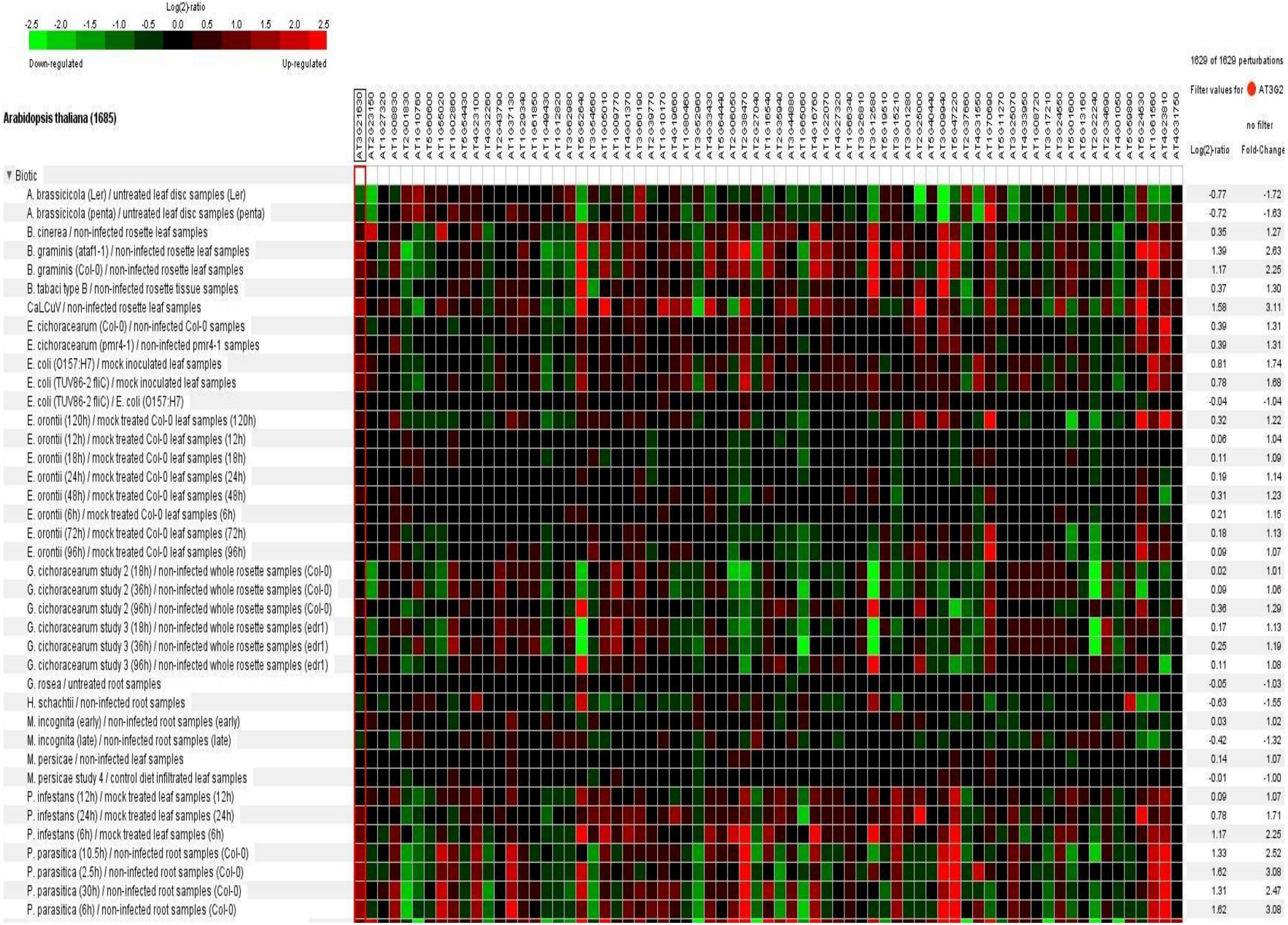

Continued

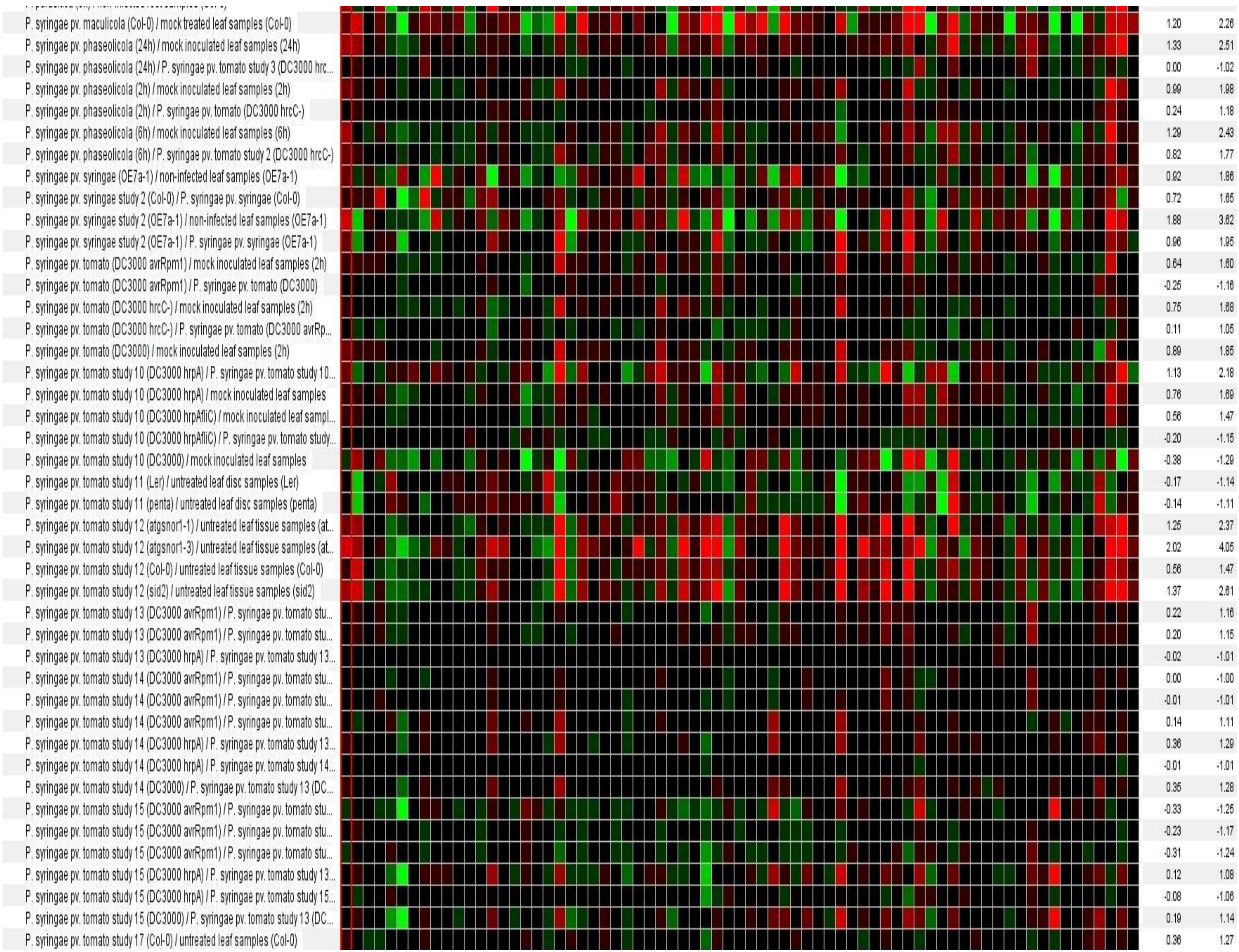
P. syringae pv. maculicola (Col-0) / mock treated leaf samples (Col-0) 1.20 2.26
P. syringae pv. phaseolicola (24h) / mock inoculated leaf samples (24h) 1.33 2.51
P. syringae pv. phaseolicola (24h) / P. syringae pv. tomato study 3 (DC3000 hrc... 0.00 -1.02
P. syringae pv. phaseolicola (2h) / mock inoculated leaf samples (2h) 0.99 1.98
P. syringae pv. phaseolicola (2h) / P. syringae pv. tomato (DC3000 hrcC-) 0.24 1.18
P. syringae pv. phaseolicola (6h) / mock inoculated leaf samples (6h) 1.29 2.43
P. syringae pv. phaseolicola (6h) / P. syringae pv. tomato study 2 (DC3000 hrcC-) 0.82 1.77
P. syringae pv. syringae (OE7a-1) / non-infected leaf samples (OE7a-1) 0.92 1.88
P. syringae pv. syringae study 2 (Col-0) / P. syringae pv. syringae (Col-0) 0.72 1.65
P. syringae pv. syringae study 2 (OE7a-1) / non-infected leaf samples (OE7a-1) 1.88 3.62
P. syringae pv. syringae study 2 (OE7a-1) / P. syringae pv. syringae (OE7a-1) 0.96 1.95
P. syringae pv. tomato (DC3000 avrRpm1) / mock inoculated leaf samples (2h) 0.64 1.60
P. syringae pv. tomato (DC3000 avrRpm1) / P. syringae pv. tomato (DC3000) -0.25 -1.16
P. syringae pv. tomato (DC3000 hrcC-) / mock inoculated leaf samples (2h) 0.75 1.68
P. syringae pv. tomato (DC3000 hrcC-) / P. syringae pv. tomato (DC3000 avrRp... 0.11 1.05
P. syringae pv. tomato (DC3000) / mock inoculated leaf samples (2h) 0.89 1.85
P. syringae pv. tomato study 10 (DC3000 hrpA) / P. syringae pv. tomato study 10... 1.13 2.18
P. syringae pv. tomato study 10 (DC3000 hrpA) / mock inoculated leaf samples 0.76 1.69
P. syringae pv. tomato study 10 (DC3000 hrpAfliC) / mock inoculated leaf sampl... 0.56 1.47
P. syringae pv. tomato study 10 (DC3000 hrpAfliC) / P. syringae pv. tomato study... -0.20 -1.15
P. syringae pv. tomato study 10 (DC3000) / mock inoculated leaf samples -0.38 -1.29
P. syringae pv. tomato study 11 (Ler) / untreated leaf disc samples (Ler) -0.17 -1.14
P. syringae pv. tomato study 11 (penta) / untreated leaf disc samples (penta) -0.14 -1.11
P. syringae pv. tomato study 12 (atgsnor1-1) / untreated leaf tissue samples (at... 1.25 2.37
P. syringae pv. tomato study 12 (atgsnor1-3) / untreated leaf tissue samples (at... 2.02 4.05
P. syringae pv. tomato study 12 (Col-0) / untreated leaf tissue samples (Col-0) 0.56 1.47
P. syringae pv. tomato study 12 (sid2) / untreated leaf tissue samples (sid2) 1.37 2.61
P. syringae pv. tomato study 13 (DC3000 avrRpm1) / P. syringae pv. tomato stu... 0.22 1.16
P. syringae pv. tomato study 13 (DC3000 avrRpm1) / P. syringae pv. tomato stu... 0.20 1.15
P. syringae pv. tomato study 13 (DC3000 hrpA) / P. syringae pv. tomato study 13... -0.02 -1.01
P. syringae pv. tomato study 14 (DC3000 avrRpm1) / P. syringae pv. tomato stu... 0.00 -1.00
P. syringae pv. tomato study 14 (DC3000 avrRpm1) / P. syringae pv. tomato stu... -0.01 -1.01
P. syringae pv. tomato study 14 (DC3000 avrRpm1) / P. syringae pv. tomato stu... 0.14 1.11
P. syringae pv. tomato study 14 (DC3000 hrpA) / P. syringae pv. tomato study 13... 0.36 1.29
P. syringae pv. tomato study 14 (DC3000 hrpA) / P. syringae pv. tomato study 14... -0.01 -1.01
P. syringae pv. tomato study 14 (DC3000) / P. syringae pv. tomato study 13 (DC... 0.35 1.28
P. syringae pv. tomato study 15 (DC3000 avrRpm1) / P. syringae pv. tomato stu... -0.33 -1.25
P. syringae pv. tomato study 15 (DC3000 avrRpm1) / P. syringae pv. tomato stu... -0.23 -1.17
P. syringae pv. tomato study 15 (DC3000 avrRpm1) / P. syringae pv. tomato stu... -0.31 -1.24
P. syringae pv. tomato study 15 (DC3000 hrpA) / P. syringae pv. tomato study 13... 0.12 1.08
P. syringae pv. tomato study 15 (DC3000 hrpA) / P. syringae pv. tomato study 15... -0.08 -1.06
P. syringae pv. tomato study 15 (DC3000) / P. syringae pv. tomato study 13 (DC... 0.19 1.14
P. syringae pv. tomato study 17 (Col-0) / untreated leaf samples (Col-0) 0.36 1.27

Continued

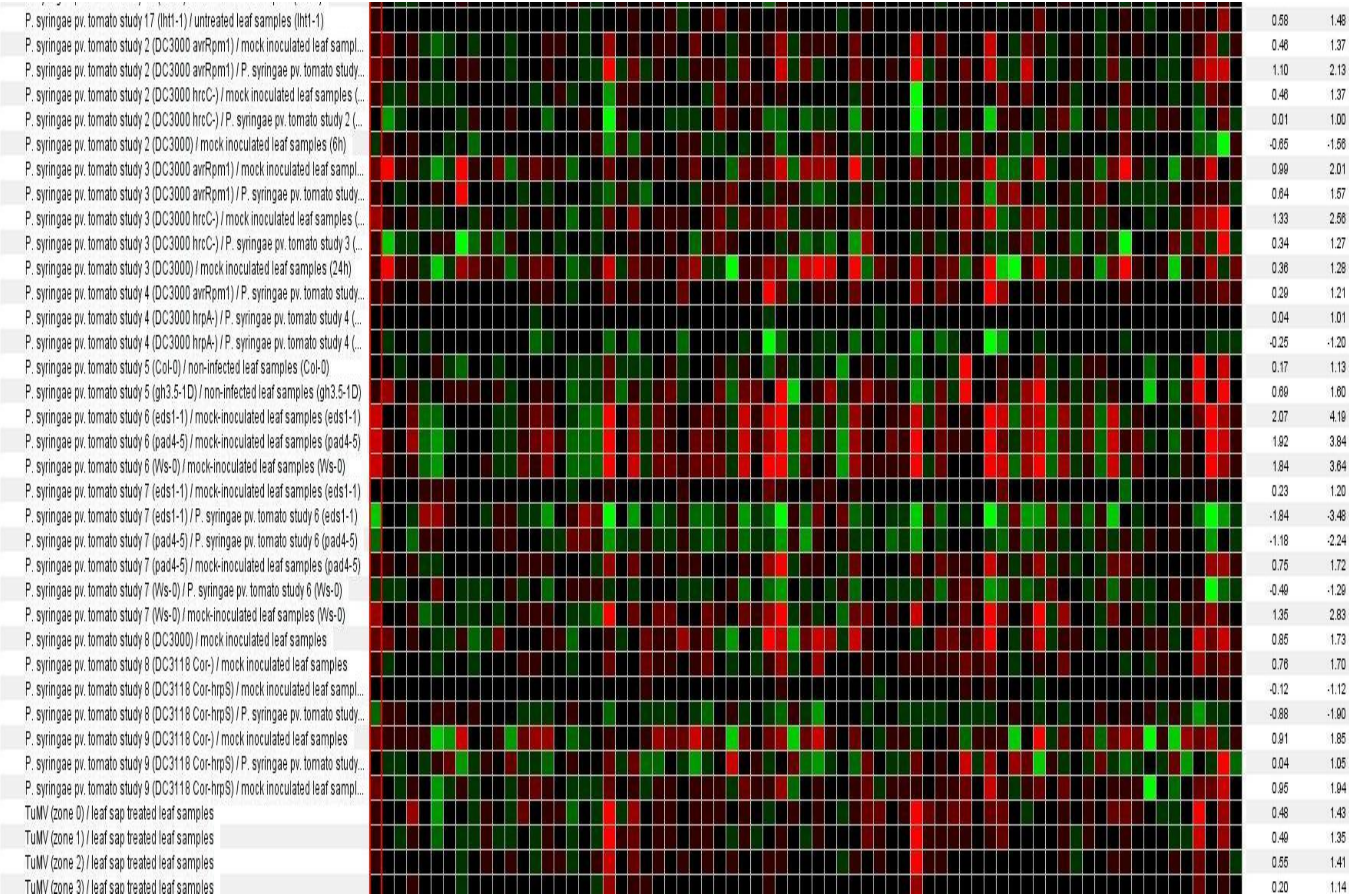

Different pathogens that influence the expression pattern of aphid and whitefly induced genes.
