# Supplementary material for: Comparative transcriptome analysis of Gossypium hirsutum L. in response to sap sucking insects: aphid and whitefly
Source: BMC Genomics. 2013 Apr 11;14:241. doi: 10.1186/1471-2164-14-241 (PMC3637549; doi:10.1186/1471-2164-14-241)
Supplement: Additional file 12 — Expression pattern by qRT-PCR of selected constant expressive contigs. Validation of transcriptome sequencing data by qRT-PCR of selected contigs that have constant expression throughout the experiment. [file 1471-2164-14-241-S12.pdf]

Additional file 12

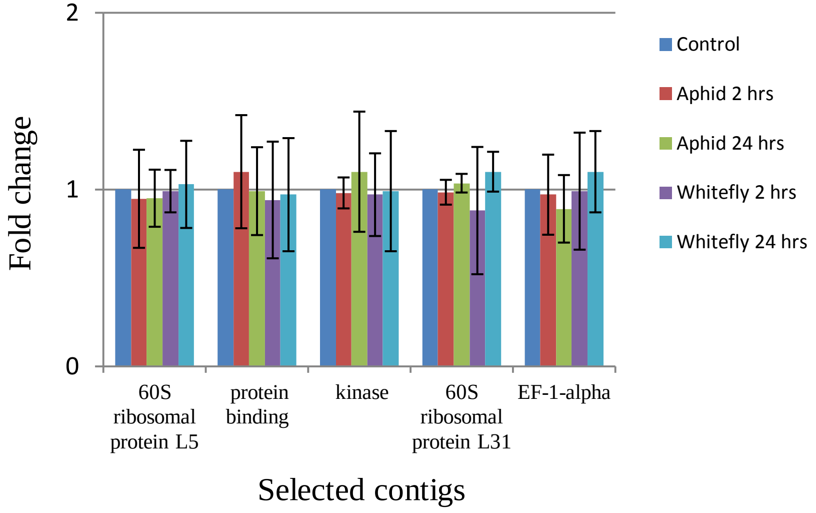

Validation of transcriptome sequencing data by qRT-PCR of selected contigs that have constant expression throughout the experiment.
